# Prevalence of intestinal protozoan parasites among Asian schoolchildren: a systematic review and meta-analysis

**DOI:** 10.1007/s15010-024-02339-1

**Published:** 2024-07-09

**Authors:** Amir Abdoli, Meysam Olfatifar, Aida Vafae Eslahi, Zeinab Moghadamizad, Oskar Nowak, Majid Pirestani, Amir Karimipour-saryazdi, Milad Badri, Panagiotis Karanis

**Affiliations:** 1 Zoonoses Research Center, Jahrom University of Medical Sciences, Jahrom, Iran; 2 Department of Parasitology and Mycology, Jahrom University of Medical Sciences, Jahrom, Iran; 3 Gastroenterology and Hepatology Diseases Research Center, Qom University of Medical Sciences, Qom, Iran; 4 Medical Microbiology Research Center, Qazvin University of Medical Sciences, Qazvin, Iran; 5 Department of Parasitology, Faculty of Medical Sciences, Tarbiat Modares University, Tehran, Iran; 6 Institute of Human Biology and Evolution, Faculty of Biology, Adam Mickiewicz University, Poznań, Poland; 7 Medical Faculty and University Hospital, University of Cologne, Cologne, Germany; 8 Department of Basic and Clinical Sciences, Medical School, Anatomy Centre, University of Nicosia, Nicosia, Cyprus

**Keywords:** Intestinal protozoan parasites, Schoolchildren, Asia, Prevalence, Meta-analysis

## Abstract

**Purpose:**

Intestinal protozoan parasites among Asian schoolchildren are a subject of concern due to their prevalence and potential health impact. Understanding and addressing this issue is crucial for public health in the region.

**Methods:**

We conducted a comprehensive search for articles published up to December 2023 across four databases, including Scopus, PubMed, ProQuest, and Web of Science. To estimate the combined prevalence, a random-effects model with a 95% confidence interval (CI) was applied, and the statistical analysis was performed using meta-analysis packages in R version (3.6.1). This study is registered with PROSPERO (CRD42023481146).

**Results:**

Among 131 eligible articles, the prevalence of intestinal protozoan parasites was 0.208 (95% CI = 0.180–0.238). Lebanon and Tajikistan had the highest country-level prevalence at 0.851 and 0.836, respectively, with *Giardia duodenalis* being the most prevalent species at 0.082.

**Conclusion:**

In summary, our study highlights the urgent public health issue of protozoan parasites among Asian schoolchildren due to poor sanitation and water quality. Immediate interventions are essential, considering climate and socioeconomic factors, to combat these infections and improve overall health.

**Supplementary Information:**

The online version contains supplementary material available at 10.1007/s15010-024-02339-1.

## Introduction

Intestinal parasites pose a significant health challenge in developing nations, where they cause substantial morbidity and mortality rates. Among those most severely impacted are children from economically disadvantaged families. It is noteworthy that more than 267 million preschool-aged children and 568 million school-aged children reside in areas characterized by the widespread and intense transmission of these parasites [1–3].

These children frequently find themselves in situations where they need the means to obtain uncontaminated and safe drinking water, proper sanitation facilities, or even basic toilets in their homes. This leaves them exceptionally susceptible to various health risks and challenges [4, 5].

On a global scale, the prevalence of intestinal protozoan parasites (IPPs) affects nearly 3.5 billion individuals, highlighting the extent of this problem. Furthermore, these parasites contribute significantly to the high annual incidence of over one billion cases of diarrheal illnesses [6].

These parasites are ubiquitous in regions characterized by tropical and subtropical climates, which often include countries in the developing world. In such areas, the environmental conditions are conducive to the proliferation of these parasites, making them a significant health concern. The challenges posed by these infections are particularly pronounced in developing nations, where limited access to clean water, sanitation facilities, and healthcare resources can exacerbate the impact of these parasites on the population's health and well-being [4, 7, 8].

Among intestinal protozoan parasites, *Cryptosporidium* spp., *Entamoeba histolytica*, and *Giardia duodenalis* are the predominant sources of infections in the human population [9, 10].

The symptomatic infection manifests as diarrhea, abdominal discomfort, and malabsorption, resulting in malnutrition and weight reduction, especially in children [11].

The most commonly utilized methods for detecting IPPs from stool samples include direct smear microscopy and various concentration techniques [12].

Nowadays, more precise methods such as copro-parasitological exams and DNA-based techniques are employed for more accurate identification of these infections [13].

In recent years, an escalating apprehension has arisen regarding the welfare of children in Asia, with a specific focus on the menace of parasitic infections. The presence of IPPs represents a significant public health challenge, emphasizing the need for a comprehensive understanding of their prevalence and geographical distribution among preschool and school-age children. These insights are essential for crafting precise and effective intervention strategies. The primary aim of this systematic review and meta-analysis is to address the substantial gap in our current knowledge by collecting and analyzing existing data to shed light on the current situation.

## Methods

### Search strategy

The current study followed the guidelines outlined by the Preferred Reporting Items for Systematic Reviews and Meta-Analysis (PRISMA) [14]. We conducted an extensive search across four databases (including PubMed, Scopus, Web of Science, and ProQuest) to retrieve relevant papers published up to December 2023, without time limitation (Supplementary Table 1). Our search employed terms related to the prevalence, frequency, epidemiology, incidence, parasitic diseases, parasites, parasitic infections, protozoan parasites, protozoan infections, protozoan diseases, protozoan pathogens, intestinal protozoans, preschool and/or school-age children, and Asia, using both AND and/or Boolean operators. The names of the 48 countries in Asia were also included in the search terms list.

Duplicate papers were automatically excluded using EndNote software X9 version. Additionally, we manually scrutinized the reference lists to identify any pertinent studies that may not have been accessible through the database search. Two authors independently conducted the searches, evaluated titles and abstracts, and thoroughly reviewed the full-text articles.

### Inclusion and exclusion criteria

Full-text articles were considered eligible upon satisfying the inclusion criteria outlined below:

1. They included cross-sectional research that reported the presence of IPPs among Asian schoolchildren.
2. They were original articles published in peer-reviewed journals.
3. Both the full-text and abstract of the articles were available in English.
4. The articles provided information on the total sample size and the precise number of individuals who tested positive for intestinal protozoan parasites.

Studies that fell into the category of case series, case reports, letters, editorials, publications without original data, review articles, articles with inconclusive findings, non-English-language publications, and studies reporting IPPs in samples from sources other than human subjects were excluded from the analysis conducted in this study. Microsoft Excel® version 2016 was used to systematically gather the following data from the included articles: author names, publication year, climate, annual precipitation, humidity levels, annual rainfall, average temperature, gender, educational status, the Global Burden of Disease (GBD) regions, district/city/province, age, mean age, income level, source of sample, diagnostic method, and type of IPPs (Tables 1, 2, 3).

**Table 1:** Main characteristics of the included studies reporting the prevalence of intestinal protozoan parasites among Asian schoolchildren

Study No	Author	Year of publication	Study Year	Country	District/City/province	Diagnostic method	Sample size	Infected	Age of selected population	Mean age	Species of protozoan	Digital Object Identifier (DOI)
1	Khudruj	2000	–	Palestine	Qalqilia	Direct smear & Concentration (Flotation)	1329	186	6–12	–	*Entamoeba histolytica / Entamoeba dispar & Giardia lamblia*	N/A
2	LEE et al	2000	–	Philippines	Legaspi	Concentration (Sedimentation)	64	30	–	–	*Giardia lamblia & Entamoeba coli & Iodamoeba butschlii & Endolimax nana*	N/A
3	Rafiei et al	2000	–	Iran	Tehran	Direct smear	1115	163	6–11	–	*Giardia lamblia*	N/A
4	Orabi	2000		Palestine	Nablus	Direct smear & Concentration (Sedimentation)	220	76	1–6	–	*Entamoeba histolytica / Entamoeba dispar & Giardia lamblia*	N/A
5	Yong et al	2000	1999	Nepal	Baharatpur	Concentration (Sedimentation)	300	123	–	–	*Entamoeba histolytica / Entamoeba dispar & Entamoeba coli & Iodamoeba butschlii & Endolimax nana & Giardia lamblia*	N/A
6	Shahabi	2000	2000–2001	Iran	Shahriar	Direct smear & Concentration (Sedimentation)	1902	621	6–15	9.3	*Entamoeba histolytica / Entamoeba dispar & Giardia lamblia*	N/A
7	Chandrasena et al	2000		Sri Lanka	Mahiyangana	Direct smear	145	44	6–15	8.3	*Entamoeba coli & Giardia lamblia & Blastocystis hominis*	N/A
8	Saifi et al	2001	2000	India	Budaun	Direct smear & Concentration (Flotation)	367	81	5–13	–	*Entamoeba histolytica / Entamoeba dispar & Giardia lamblia*	N/A
9	Uga et al	2002	2000	Indonesia	Bekasi	Concentration (Flotation)	285	1	14	–	*Giardia lamblia*	N/A
10	LEE et al	2002	-	Cambodia	Kampongcham	Concentration (Sedimentation)	251	37	–	–	*Entamoeba histolytica / Entamoeba dispar & Giardia lamblia & Entamoeba coli & Iodamoeba butschlii*	N/A
11	Waikagul et al	2002	-	Thailand	Nan	Concentration (Sedimentation)	1010	366	–	–	*Entamoeba histolytica / Entamoeba dispar & Giardia lamblia & Entamoeba coli & Iodamoeba butschlii & Chilomastix mesnili & Endolimax nana*	N/A
12	Monawar Hosain et al	2003	1999	Bangladesh	Sherpur	Direct smear	149	56	5–13	–	*Entamoeba histolytica / Entamoeba dispar & Giardia lamblia*	N/A
13	Piangjai et al	2003	1997–1998	Thailand	Chiang Mal	Concentration (Sedimentation)	403	192	–	–	*Entamoeba histolytica / Entamoeba dispar & Giardia lamblia & Entamoeba coli & Sarcocystis spp & Chilomastix mesnili & Endolimax nana & Blastocystis hominis*	N/A
14	Chhetri et al	2003	2003	Nepal	Bhaktapur	Direct smear	593	41	6–15	–	*Giardia lamblia*	N/A
15	Ahmed Zakai	2004	-	Saudi Arabia	Jeddah	Concentration (Sedimentation)	231	21	6–14	–	*Giardia lamblia & Entamoeba coli & Iodamoeba butschlii & Chilomastix mesnili*	N/A
16	Nematian et al	2004	1998	Iran	Tehran	Direct smear & Concentration (Sedimentation)	19,209	2882	–	8.5 ± 1.5	*Entamoeba histolytica / Entamoeba dispar & Giardia lamblia & Entamoeba coli & Blastocystis hominis*	10.1016/j.actatropica.2004.06.010
17	Astal	2004	2002–2003	Palestine	Khan Younis	Direct smear & Concentration (Flotation & Sedimentation)	1370	256	6–11	–	*Entamoeba histolytica / Entamoeba dispar & Giardia lamblia & Entamoeba coli*	10.1007/s00436-004–1234-1
18	Okyay et al	2004	-	Turkey	Aydin	Direct smear & Concentration (Sedimentation)	456	49	–	10.34 ± 2.27	*Giardia lamblia & Entamoeba coli*	10.1186/1471–2458-4–64
19	Krishna Sharma et al	2004	-	Nepal	Kathmandu	Direct smear & Concentration (Sedimentation)	533	145	4–19	-	*Entamoeba histolytica / Entamoeba dispar & Giardia lamblia & Entamoeba coli & Entamoeba hartmanni & Iodamoeba butschlii*	N/A
20	Saksirisampant et al	2004	2002–2003	Thailand	Chiang Mai	Concentration (Sedimentation)	542	191	6–19	–	*Entamoeba histolytica / Entamoeba dispar & Giardia lamblia & Entamoeba coli & Endolimax nana & Iodamoeba butschlii & Chilomastix mesnili*	N/A
21	Daryani et al	2005	2003	Iran	Ardabil	Direct smear & Concentration (Sedimentation)	1070	319	7–13	–	*Giardia lamblia & Entamoeba coli & Iodamoeba butschlii & Blastocystis hominis*	N/A
22	Chandrashekhar et al	2005	2004	Nepal	-	Direct smear	2091	313		8.8	*Entamoeba histolytica / Entamoeba dispar & Giardia lamblia*	N/A
23	Sadjjadi et al	2005	-	Iran	Marvdasht	Direct smear & Concentration (Sedimentation)	337	103	3–6	–	*Entamoeba histolytica / Entamoeba dispar & Giardia lamblia & Iodamoeba butschlii & Blastocystis hominis*	N/A
24	Uga et al	2005	2003–2004	Vietnam	Hanoi	Direct smear & Concentration (Sedimentation)	217	22	14–15	–	*Entamoeba histolytica / Entamoeba dispar & Giardia lamblia & Entamoeba coli*	N/A
25	Wongjindanon et al	2005	2002–2004	Thailand	Samut Sakhon	Direct smear	4014	118	5–7	–	*Giardia lamblia*	N/A
26	Chhakda et al	2006	1998–1999	Cambodia	–	Concentration (Sedimentation)	789	147	–	11.3	*Entamoeba histolytica / Entamoeba dispar & Giardia lamblia*	N/A
27	Kanoa et al	2006	2003	Palestine	Gaza	Direct smear & Staining	432	64	6–11	–	*Entamoeba histolytica / Entamoeba dispar & Giardia lamblia*	N/A
28	Patel et al	2006	2004–2005	Oman	Dahahira	Direct smear	436	157	9–10	–	*Entamoeba histolytica / Entamoeba dispar & Giardia lamblia & Entamoeba coli*	N/A
29	Saksirisampant et al	2006	2004	Thailand	–	Concentration (Sedimentation)	1037	37	3–12	–	*Giardia lamblia & Entamoeba coli & Blastocystis hominis & Endolimax nana*	N/A
30	Yaicharoen et al	2006	2004	Thailand	Nakhon Pathom	Direct smear & Culture	814	103	7–13	–	*Giardia lamblia & Blastocystis hominis*	N/A
31	Aksoy et al	2007	2003	Turkey	Izmir	Direct smear & Concentration (Sedimentation) & Staining	1127	305	7–14	–	*Entamoeba histolytica / Entamoeba dispar & Giardia lamblia & Blastocystis hominis & Entamoeba coli & Endolimax nana & Entamoeba hartmanni & Iodamoeba butschlii*	N/A
32	Aminzadeh et al	2007	2000–2001	Iran	Varamin	Direct smear & Concentration (Sedimentation)	293	110	–	–	*Giardia lamblia & Entamoeba coli & Blastocystis hominis & Endolimax nana & Iodamoeba butschlii*	N/A
33	Ngrenngarmlert et al	2007	2004	Thailand	Nakhon Pathom	Direct smear & Concentration (Sedimentation)	1920	205	7–12	–	*Entamoeba histolytica / Entamoeba dispar & Giardia lamblia & Entamoeba coli & Endolimax nana & Blastocystis hominis*	N/A
34	Hafiz Mahsol et al	2008	2005–2006	Malaysia	Kota Kinabalu	Direct smear & Concentration (Sedimentation)	100	60	7–9	–	*Entamoeba histolytica / Entamoeba dispar & Giardia lamblia & Entamoeba coli & Endolimax nana & Entamoeba hartmanni & Iodamoeba butschlii*	N/A
35	Nematian et al	2008	–	Iran	Teharan	Direct smear & Concentration (Sedimentation)	19,209	3147	–	8.5	*Entamoeba histolytica / Entamoeba dispar & Giardia lamblia & Entamoeba coli & Blastocystis hominis*	10.1179/136485908X267876
36	Almeire et al	2008	2006	Syria	Damascus	Direct smear	1469	341	6–12	–	*Entamoeba histolytica / Entamoeba dispar & Giardia lamblia & Entamoeba coli & Endolimax nana & Entamoeba hartmanni & Iodamoeba butschlii & Chilomastix mesnili & Blastocystis hominis*	N/A
37	Gyawali et al	2009	2007–2008	Nepal	Dharan	Direct smear & Concentration (Sedimentation)	182	31	4–10	–	*Entamoeba histolytica / Entamoeba dispar & Giardia lamblia*	N/A
38	Al-Shamiri et al	2010	2006–2007	Yemen	Taiz	Direct smear & Concentration (Flotation) & Staining	712	247	0–12	–	*Cryptosporidium spp*	N/A
39	Aly et al	2010	2009	Saudi Arabia	Tabuk	Direct smear & Concentration (Flotation & Sedimentation) & Staining	812	59	2–12	–	*Entamoeba histolytica / Entamoeba dispar & Giardia lamblia & Cryptosporidium spp*	N/A
40	Sehgal et al	2010	2007	India	Chandigarh	Direct smear	360	132		–	*Entamoeba histolytica / Entamoeba dispar & Giardia lamblia & Entamoeba coli*	N/A
41	Singh et al	2010	2002–2003	India	Srinagar	Direct smear	514	36	5–14	–	*Giardia lamblia*	N/A
42	Rayan et al	2010		India	–	Direct smear	195	60	5–11	–	*Entamoeba histolytica / Entamoeba dispar & Giardia lamblia & Entamoeba coli & Blastocystis hominis & Iodamoeba butschlii*	10.4103/0377–4929.68292
43	Matthys et al	2011	2009	Tajikistan	–	Concentration (Sedimentation)	594	497	7–11	–	*Entamoeba histolytica / Entamoeba dispar & Giardia lamblia & Entamoeba coli & Endolimax nana & Entamoeba hartmanni & Iodamoeba butschlii & Chilomastix mesnili & Blastocystis hominis*	10.1186/1756–3305-4–195
44	S. Hussein	2011	2008	Palestine	Nablus	Direct smear & Concentration (Sedimentation) & Staining & PCR	735	101	7–13	9.5	*Entamoeba histolytica / Entamoeba dispar & Giardia lamblia*	10.1111/j.1365–3156.2010.02674.x
45	Saeed Jaeffer	2011	2009–2010	Iraq	Baghdad	Direct smear & Concentration (Sedimentation)	513	70	0–12	-	*Entamoeba histolytica / Entamoeba dispar & Giardia lamblia*	N/A
46	Taheri et al	2011	2007	Iran	–	Direct smear & Concentration (Sedimentation)	2169	1175	–	8.4	*Giardia lamblia & Chilomastix mesnili & Trichomonas intestinalis & Entamoeba coli*	N/A
47	Bhandari et al	2011	2008–2009	Nepal	Kavrepalanchowk	Direct smear & Concentration (Sedimentation)	360	21	–	–	*Giardia lamblia*	N/A
48	Aher et al	2011	–	India	Loni	Direct smear	624	136	6–12	–	*Entamoeba histolytica / Entamoeba dispar & Giardia lamblia & Iodamoeba butschlii & Blastocystis hominis & Entamoeba coli*	N/A
49	Abdulsalam et al	2012	2010	Malaysia	–	Direct smear & culture	300	136	6–12	9	*Entamoeba histolytica / Entamoeba dispar & Giardia lamblia & Blastocystis hominis*	10.1017/S0031182012000340
50	Daryani et al	2012	2009–2010	Iran	Sari	Direct smear & Concentration (Sedimentation) & Staining	1100	256	7–14	10.62 ± 2.34	*Giardia lamblia & Entamoeba coli & Endolimax nana & Blastocystis hominis*	10.1016/j.trstmh.2012.05.010
51	Mukhiya et al	2012	2011	Nepal	Sindhuli	Direct smear & Concentration (Sedimentation)	342	44		–	*Entamoeba histolytica / Entamoeba dispar & Giardia lamblia & Blastocystis hominis & Endolimax nana & Entamoeba coli*	N/A
52	Panda et al	2012	2008–2009	India	Nellimarla Mandal	Direct smear	124	52	6–9	–	*Entamoeba histolytica / Entamoeba dispar & Giardia lamblia*	N/A
53	Rostami et al	2012	2010–2011	Iran	Golestan	Direct smear & Concentration (Sedimentation)	800	209	8–12	8.5	*Entamoeba histolytica / Entamoeba dispar & Giardia lamblia & Blastocystis hominis*	10.3923/pjbs.2012.1119.1125
54	Shrestha et al	2012	2010–2011	Nepal	Baglung	Direct smear & Concentration (Sedimentation)	260	40	≤ 4 –10	–	*Entamoeba histolytica / Entamoeba dispar & Giardia lamblia & Entamoeba coli*	N/A
55	Shoaib Khan et al	2012	2010–2011	Pakistan	Banuu	Direct smear	100	9	5–10	–	*Entamoeba histolytica / Entamoeba dispar & Giardia lamblia*	N/A
56	Al–Mekhlafi et al	2013	2010	Malaysia	Pahang	Direct smear & Concentration (Sedimentation) & Staining	374	83	7–12	–	*Giardia lamblia*	10.1371/journal.pntd.0002516
57	Bilakshan Sah et al	2013	2011–2012	Nepal	Itahari	Direct smear	200	37	12–15	–	*Entamoeba histolytica / Entamoeba dispar & Giardia lamblia*	10.4103/2229–5070.122143
58	Singh Khadka et al	2013	2011–2012	Nepal	Pokhara	Direct smear & Concentration (Sedimentation)	100	9	3–15	–	*Entamoeba histolytica / Entamoeba dispar & Giardia lamblia*	N/A
59	J. Lakhani et al	2013	–	India	Vadodara	Direct smear & Concentration (Sedimentation)	140	28	6–12	–	*Entamoeba histolytica / Entamoeba dispar & Giardia lamblia & Entamoeba coli*	N/A
60	Kitvatanachai et al	2013	2010	Thailand	Muang Pathum Thani	Direct smear & Concentration (Sedimentation)	202	21	7–12	–	*Entamoeba histolytica / Entamoeba dispar & Giardia lamblia & Entamoeba coli & Endolimax nana*	10.1016/S1995–7645(13)60,121–2
61	Ashok et al	2013	2006	India	Amalapuram	Direct smear & Concentration (Sedimentation)	208	81		8.8 ± 2.11	*Entamoeba histolytica / Entamoeba dispar & Giardia lamblia & Entamoeba coli*	10.17795/semj16652
62	Tandukar et al	2013	2011	Nepal	Lalitpur	Direct smear & Concentration (Sedimentation) & Staining	1392	189	5–15	–	*Entamoeba histolytica / Entamoeba dispar & Giardia lamblia & Entamoeba coli & Cyclospora spp & Blastocystis hominis*	10.1186/1756–0500-6–449
63	Sah et al	2013	2011–2012	Nepal	Itahari	Direct smear	200	37	12–15	–	*Entamoeba histolytica / Entamoeba dispar & Giardia lamblia*	N/A
64	Raj Tiwari et al	2013	2009–2010	Nepal	Dadeldhura	Direct smear	530	13	4–12	–	*Giardia lamblia*	N/A
65	Yadav et al	2013	2012	Nepal	Dhnusha	Staining	500	64	3–14	–	Cryptosporidium spp	N/A
66	Ullah et al	2014	2010	Pakistan	Khyber Pakhtunkhwa	Direct smear & Staining	222	20	4–15	–	*Entamoeba histolytica / Entamoeba dispar & Giardia lamblia & Entamoeba coli*	10.12692/ijb/5.1.1–8
67	Pradhan et al	2014	2012	Nepal	Kathmandu	Direct smear	194	34	6–10	–	*Entamoeba histolytica / Entamoeba dispar & Giardia lamblia*	N/A
68	Padmaja et al	2014	2013–2014	India	Amalapuram	Direct smear & Concentration (Sedimentation)	200	67	–	–	*Entamoeba histolytica / Entamoeba dispar & Giardia lamblia*	N/A
69	Kiran et al	2014	2013	India	Bhopal	Direct smear	300	84	6–12	–	*Entamoeba histolytica / Entamoeba dispar & Giardia lamblia*	N/A
70	Bilakshan Sah et al	2014	2007–2008	Nepal	Dharan	Direct smear	935	121	–	–	*Entamoeba histolytica / Entamoeba dispar & Giardia lamblia*	N/A
71	Jaiswal et al	2014	2012–2013	Nepal	Kaski	Direct smear	163	12	3–15	–	*Entamoeba histolytica / Entamoeba dispar & Giardia lamblia*	N/A
72	Al–Delaimy et al	2014	2012	Malaysia	Pahang	Direct smear & Concentration (Sedimentation) & Staining	498	237	6–12	–	*Entamoeba histolytica / Entamoeba dispar & Giardia lamblia & Cryptosporidium spp*	10.1371/journal.pntd.0003074
73	Pandey et al	2015	2014	Nepal	Kathmandu	Direct smear & Concentration (Sedimentation)	300	9	–	–	*Entamoeba histolytica / Entamoeba dispar & Giardia lamblia*	10.1016/S2222–1808(15)60,864–7
74	Polseela et al	2015	2009–2010	Thailand	Phitsanulok	Direct smear & Concentration (Sedimentation)	352	4	7–15	–	*Entamoeba coli* & *Giardia lamblia*	10.1016/S2222–1808(15)60,832–5
75	Altınoz Aytar et al	2015	2009–2012	Turkey	Yıgılca	Direct smear & Staining	523	79	–	–	*Giardia lamblia*	N/A
76	Bhattachan et al	2015	2012	Nepal	Kathmandu	Direct smear & Concentration (Sedimentation)	296	41	0–18	–	*Entamoeba histolytica / Entamoeba dispar & Giardia lamblia & Entamoeba coli & Endolimax nana*	N/A
77	Bhandari et al	2015	2014	Nepal	Kathmandu	Direct smear & Concentration (Sedimentation) & Staining	507	77	3–14	–	*Entamoeba histolytica / Entamoeba dispar & Giardia lamblia & Entamoeba coli &* Cryptosporidium spp & *Blastocystis hominis*	N/A
78	Yadav et al	2016	2014	Nepal	Kathmandu	Direct smear & Staining	507	169	6–10	–	*Entamoeba histolytica / Entamoeba dispar & Giardia lamblia &* Cryptosporidium spp	N/A
79	Shrestha et al	2016	2013	Nepal	Bhaktapur	Direct smear & Concentration (Sedimentation)	184	50	2–17	–	*Entamoeba histolytica / Entamoeba dispar & Giardia lamblia & Entamoeba coli & Cyclospora spp & Blastocystis hominis*	N/A
80	Sherchand et al	2016		Nepal	Kathmandu	Direct smear & Concentration (Flotation & Sedimentation) & Staining	187	55	3–12	–	*Cryptosporidium spp*	N/A
81	Dhital et al	2016	2013	Nepal	Kathmandu	Direct smear & Concentration (Sedimentation) & Staining	600	136	3–15	–	*Entamoeba histolytica / Entamoeba dispar & Giardia lamblia & Entamoeba coli & cyclospora & Blastocystis hominis & Cryptosporidium spp*	10.1186/s40064–016–3477–6
82	Doi et al	2016	2013–2014	Thailand	Sakon Nakhon	Concentration (Sedimentation)	417	42	4–12	–	*Entamoeba histolytica / Entamoeba dispar & Giardia lamblia*	N/A
83	Osman et al	2016	2013	Lebanon	Tripoli	Direct smear & Staining & PCR	249	212	3–16	10.3 ± 2.7	*Giardia lamblia & Blastocystis hominis & Dientamoeba fragilis & Cryptosporidium spp*	10.1371/journal.pntd.0004496
84	Arıkan et al	2016	2014	Turkey	Kutahya	Direct smear & Concentration (Sedimentation) & Staining	471	29	5–11	7.91 ± 1.4	*Entamoeba histolytica / Entamoeba dispar & Giardia lamblia & Blastocystis hominis & Entamoeba coli*	10.21101/cejph.a4231
85	Nithyamathi et al	2016	2012–2013	Malaysia	–	Direct smear & Concentration (Sedimentation) & Culture	1760	188	7–12	–	*Giardia lamblia & Blastocystis hominis*	10.1371/journal.pone.0136709
86	Korzeniewski et al	2016	2013–2014	Afghanistan	–	Direct smear & Concentration (Sedimentation)	500	97	7–18	–	*Entamoeba histolytica / Entamoeba dispar & Giardia lamblia*	10.5604/12321966.1226864
87	Khanal et al	2016	2015	Nepal	Lumbini	Direct smear & Concentration (Sedimentation)	217	2	4–15	–	*Entamoeba histolytica / Entamoeba dispar & Giardia lamblia*	N/A
88	R. Alsubaie et al	2016	2010	Yemen	Ibb	Direct smear & Concentration (Sedimentation)	258	148	8–15	–	*Entamoeba histolytica / Entamoeba dispar & Giardia lamblia*	10.1016/j.jtumed.2015.10.006
89	Al–Mekhlaf et al	2016	2013–2015	Yemen	Sanaʽa	Direct smear & Concentration (Sedimentation)	1214	458	5–15	9.3	*Entamoeba histolytica / Entamoeba dispar & Giardia lamblia*	10.1016/j.actatropica.2016.08.009
90	Ghani et al	2016		Pakistan	Lahore	Direct smear	300	27		–	*Entamoeba histolytica / Entamoeba dispar & Giardia lamblia & Entamoeba coli*	10.5897/JPVB2016.0264
91	Zulfa et al	2017		Indonesia	Jakarta	Direct smear & Culture & PCR	156	69	6–12	–	*Blastocystis hominis*	10.1088/1742–6596/884/1/012031
92	Turki et al	2017	2016	Iran	Bandar Abbas	Direct smear & Concentration (Sedimentation) & Staining	1465	91	6–14	9.2	*Giardia lamblia & Entamoeba coli & Blastocystis hominis & Chilomastix mesnili*	10.1007/s12639–016–0862–6
93	Sankur et al	2017	2014	Turkey	Mugla	Direct smear & Culture & PCR	468	35	6–11	8.4 ± 1.2	*Blastocystis hominis*	N/A
94	Barazesh et al	2017	2011–2012	Iran	Bushehr	Direct smear & Concentration (Sedimentation)	203	39		–	*Giardia lamblia & Entamoeba coli & Blastocystis hominis & Endolimax nana*	10.17795/ajcmi–34335
95	Sari et al	2017		Indonesia	Jakarta	Direct smear & Culture & PCR	141	61		–	*Giardia lamblia & Entamoeba coli & Blastocystis hominis*	10.1093/tropej/fmx051
96	Saki et al	2017	2014	Iran	Ahvaz	Direct smear & Concentration (Sedimentation)	306	88	6–12	-	*Entamoeba histolytica / Entamoeba dispar & Giardia lamblia & Entamoeba coli & Blastocystis hominis*	10.5812/jjhr.40326
97	Rai et al	2017	2016	Nepal	Lokhim	Direct smear	359	74	4–16	–	*Entamoeba histolytica / Entamoeba dispar & Giardia lamblia*	10.15406/jmen.2017.04.00102
98	Bahmani et al	2017	2015	Iran	Sanandaj	Direct smear & Concentration (Sedimentation)	400	143	7–15	12.3	*Giardia lamblia & Entamoeba coli & Blastocystis hominis & Iodamoeba butschlii & Endolimax nana*	N/A
99	Jameel et al	2017	2015	Iraq	Zakho	Direct smear	103	8	6–12	–	*Entamoeba histolytica / Entamoeba dispar & Giardia lamblia*	N/A
100	Babakhani et al	2017	20,166	Iran	Gashki	Direct smear & Concentration (Sedimentation)	200	84	7–14	10.7 ± 2.29	*Giardia lamblia & Entamoeba coli & Blastocystis hominis & Chilomastix mesnili & Iodamoeba butschlii*	10.22038/ijp.2017.23173.1949
101	Jaiswal et al	2017	2016	Nepal	Tanahun	Direct smear & Concentration (Flotation & Sedimentation)	150	27	7–13	–	*Entamoeba histolytica / Entamoeba dispar & Giardia lamblia*	N/A
102	Tenali et al	2018	2015–2016	India	–	Direct smear & Staining	1246	173	5–18	12.6 ± 1.65	*Entamoeba histolytica / Entamoeba dispar & Giardia lamblia & Cryptosporidium spp & Isospora belli*	10.18203/2349–3291.ijcp20180001
103	Kyaw et al	2018	2018	Thailand	Ratchaburi	Direct smear & Concentration (Sedimentation)	252	36	9–17	11.86 ± 1.52	*Entamoeba histolytica / Entamoeba dispar & Giardia lamblia & Entamoeba coli & Endolimax nana & Blastocystis hominis*	N/A
104	Tandukar et al	2018	2016	Nepal	Kathmandu	Direct smear & Concentration (Sedimentation) & Staining & PCR	333	68	0–15	–	*Entamoeba histolytica / Entamoeba dispar & Giardia lamblia & Entamoeba coli & Cyclospora spp*	10.1007/s00436–017–5706–5
105	Punsawad et al	2018	2016	Thailand	Nakhon Si Thammarat	Direct smear & Concentration (Sedimentation)	299	17	7–12	–	*Giardia lamblia & Blastocystis hominis*	10.1186/s12889–018–6023–3
106	Gopalakrishnan et al	2018	2017	India	Anakaputhur	Direct smear	250	71	13–18	–	*Entamoeba histolytica / Entamoeba dispar & Giardia lamblia*	10.4103/jfmpc.jfmpc_89_18
107	Diarthini et al	2018	2016	Indonesia	Bali	Direct smear	103	29	6–13	–	*Blastocystis hominis*	N/A
108	Assavapongpaiboon et al	2018	2016	Thailand	Saraburi	Direct smear & Concentration (Sedimentation) & Culture	263	72	4–15	7.9 ± 2.5	*Entamoeba histolytica / Entamoeba dispar & Giardia lamblia & Entamoeba coli & Endolimax nana & Blastocystis hominis*	10.4269/ajtmh.17–0240
109	Bansal et al	2018	–	India	Rishikesh	Direct smear & Concentration (Sedimentation) & Staining	461	80	–	7.26 ± 1.57	*Entamoeba histolytica / Entamoeba dispar & Giardia lamblia & Cryptosporidium spp*	N/A
110	Upama KC et al	2019	–	Nepal	Kathmandu	Direct smear & Concentration (Sedimentation)	330	161	–	–	*Entamoeba histolytica / Entamoeba dispar & Giardia lamblia & Entamoeba coli & Endolimax nana & Blastocystis hominis & Entamoeba hartmanni*	10.3126/ijasbt.v7i1.21637
111	Rather et al	2019	2010	India	Kashmir	Concentration (Sedimentation)	130	11	5–16	–	*Entamoeba histolytica / Entamoeba dispar & Giardia lamblia*	N/A
112	Gurung et al	2019	2017	Nepal	Kathmandu	Direct smear & Concentration (Flotation & Sedimentation)	160	2	–	–	Entamoeba coli	10.21089/njhs.43.0097
113	Bakarman et al	2019	2015–2016	Saudi Arabia	Jeddah	Direct smear & Concentration (Sedimentation)	581	25	6–16	11.69 ± 1.84	*Giardia lamblia & Entamoeba coli & Endolimax nana & Blastocystis hominis*	10.2991/jegh.k.190219.001
114	Lubis et al	2019	2018	Indonesia	Medan	Direct smear & Staining	124	19	–	–	*Giardia lamblia & Blastocystis hominis*	10.3889/oamjms.2019.721
115	Qasem et al	2020	2018	Yemen	Ibb	Direct smear & Concentration (Sedimentation)	300	161	6–16	–	*Entamoeba histolytica / Entamoeba dispar & Giardia lamblia*	10.22270/ujpr.v5i2.388
116	Gupta et al	2020	2017	Nepal	Saptari	Direct smear & Concentration (Sedimentation)	258	86	5–15	–	*Entamoeba histolytica / Entamoeba dispar & Giardia lamblia & Entamoeba coli*	10.1186/s41182–020–00261–4
117	Alharazi et al	2020	2019	Yemen	Taiz	Direct smear & Concentration (Sedimentation)	385	71	7–15	–	*Entamoeba histolytica / Entamoeba dispar & Giardia lamblia*	10.3934/publichealth.2020059
118	Afridi et al	2021	2016–2017	Pakistan	Skardu	Direct smear & Staining	300	66	2–5	–	Cryptosporidium spp	N/A
119	Sari et al	2021	2016	Indonesia	Jakarta	Direct smear & Staining	157	52	6–11	–	*Giardia lamblia & Blastocystis hominis*	10.3889/oamjms.2021.5711
120	Sah et al	2021	2018	Nepal	Janakpurdham	Direct smear & Concentration (Sedimentation)	155	3	5–17	–	*Entamoeba histolytica / Entamoeba dispar & Giardia lamblia*	10.18231/j.ijmmtd.2021.021
121	Shrestha et al	2021	2018–2019	Nepal	Dharan	Direct smear & Concentration (Sedimentation)	400	3	6–11	–	*Entamoeba histolytica / Entamoeba dispar*	10.1155/2021/6632469
122	Wijayanti et al	2021	2019–2020	Indonesia	Boyolali	Direct smear & Staining	127	17	6–14	–	*Entamoeba histolytica / Entamoeba dispar & Entamoeba coli & Blastocystis hominis*	10.15562/bmj.v10i2.2443
123	Alharrazi	2022	2019	Yemen	Taiz	Direct smear & Concentration (Sedimentation)	478	145	6–15	–	*Entamoeba histolytica / Entamoeba dispar & Giardia lamblia*	10.2478/helm–2022–0032
124	Dahal et al	2022	2021	Nepal	Kathmandu	Direct smear	409	28	5–18	–	*Entamoeba histolytica / Entamoeba dispar & Giardia lamblia*	10.3126/nmcj.v24i2.46027
125	Edrees et al	2022	2021–2022	Yemen	Amran	Direct smear & Concentration (Sedimentation)	360	312	6–15	–	*Entamoeba histolytica / Entamoeba dispar & Giardia lamblia*	10.51610/rujms6.2.2022.135
126	Edrees et al	2022	2021	Yemen	Sana’a	Direct smear	173	62	9–13	–	*Entamoeba histolytica / Entamoeba dispar & Giardia lamblia*	N/A
127	Khan et al	2022	2016	Pakistan	Lower Dir	Direct smear & Concentration (Sedimentation)	184	1	10–17	14 ± 3.05	*Entamoeba histolytica / Entamoeba dispar*	10.1016/j.sjbs.2021.12.055
128	Salih et al	2022	2021–2022	Iraq	Duhok	Direct smear & Concentration (Sedimentation)	1172	322	6–12	–	*Entamoeba histolytica / Entamoeba dispar & Giardia lamblia & Blastocystis hominis*	10.26682/sjuod.2022.25.2.5
129	AL–Mekhlafi et al	2023	2022	Yemen	Sana’a	Direct smear & Concentration (Sedimentation) & Staining	400	131	7–12	9.52 ± 2.9	*Entamoeba histolytica / Entamoeba dispar & Giardia lamblia & Cryptosporidium spp & Cyclospora spp & Isospora belli*	10.22270/ujpr.v8i3.943
130	Karmacharya et al	2023	2019–2020	Nepal	Bhaktapur	Direct smear & Concentration (Sedimentation)	190	29	–	–	*Entamoeba histolytica / Entamoeba dispar & Giardia lamblia*	10.3126/njz.v7i1.56307
131	Subhan et al	2023	2019	Pakistan	Bajawar	Direct smear	402	92	4–12	–	*Entamoeba histolytica / Entamoeba dispar & Giardia lamblia*	10.34172/ijmpes.2023.04

Not applicable (N/A)

**Table 2:** Sub–group analysis based on annual precipitation, humidity, annual rainfall, average temperature, climate, countries, income level, diagnostic method, source of samples, GBD geographies regions, and educational level in included studies

Variables	No studies	Sample size	Infected	Pooled prevalence (95% CI)	Heterogeneity		
					*I*^*2*^	τ^2^	p-value
Annual precipitation							
< 20	39	63,883	12,887	0.2579 (0.2046–0.3151)	99	0.0400	P < .001
> 100	13	2708	584	0.2359 (0.1510–0.3328)	96	0.0370	P < .001
20–100	79	39,634	6759	0.1771 (0.1452–0.2115)	98	0.0375	P < .001
Total	131	106,225	20,230	0.2085 (0.1801–0.2383)	98	0.0426	P < .001
Humidity (%)							
< 40	24	54,098	11,165	0.3245 (0.2548–0.3983)	99	0.0362	P < .001
40–75	102	49,050	8377	0.1764 (0.1489–0.2055)	98	0.0352	P < .001
> 75	5	3077	688	0.3007 (0.1685–0.4524)	98	0.0312	P < .001
Total	131	106,225	20,230	0.2085 (0.1801–0.2383)	98	0.0426	P < .001
Annual rainfall (mm)							
< 400	36	60,971	12,407	0.2608 (0.2038–0.3222)	99	0.0391	P < .001
400–1000	15	8426	1745	0.1939 (0.0954–0.3167)	98	0.642	P < .001
1001–1500	13	11,525	1404	0.1431 (0.0688–0.2385)	99	0.0400	P < .001
> 1500	67	25,303	4674	0.1933 (0.1590–0.2300)	97	0.0325	P < .001
Total	131	106,225	20,230	0.2085 (0.1801–0.2383)	98	0.0426	P < .001
Average temperature (°C)							
10–20	70	75,938	15,074	0.2052 (0.1691–0.2439)	98	0.0381	P < .001
> 20	61	30,287	5156	0.2069 (0.1661–0.2508)	98	0.0421	P < .001
Total	131	106,225	20,230	0.2085 (0.1801–0.2383)	98	0.0426	P < .001
Climate							
Tropical rainforest climate	14	4334	1026	0.2916 (0.1929–0.4013)	98	0.0464	P < .001
Semi-desert climate	20	53,904	10,113	0.2438 (0.1930–0.2984)	99	0.0191	P < .001
Monsoon-influenced humid subtropical climate	37	14,810	2372	0.1469 (0.1109–0.1870)	97	0.0266	P < .001
Tropical wet and dry climate	14	5119	1092	0.2390 (0.1816–0.3015)	95	0.0169	P < .001
Tropical savanna climate	15	12,565	1588	0.1461 (0.0856–0.2193)	99	0.0345	P < .001
desert climate	24	11,699	3233	0.2428 (0.1635–0.3320)	98	0.0597	P < .001
Hot-summer Mediterranean climate	7	3794	806	0.2222 (0.0637–0.4407)	99	0.0996	P < .001
Total	131	106,225	20,230	0.2085 (0.1801–0.2383)	98	0.0426	P < .001
Countries							
Palestine	5	4086	683	0.1845 (0.1202–0.2589)	92	0.0100	P < .001
Philippines	1	64	30	0.4688 (0.3518–0.5893)	–	–	–
Iran	15	49,818	9430	0.2644 (0.2022–0.3318)	99	0.0207	P < .001
Nepal	35	14,444	2294	0.1431 (0.1069–0.1835)	97	0.0260	P < .001
Indonesia	7	1093	248	0.2246 (0.0983–0.3835)	97	0.0525	P < .001
Sri Lanka	1	145	44	0.3034 (0.2345–0.3826)	–	–	–
India	14	5119	1092	0.2390 (0.1816–0.3015)	95	0.0169	P < .001
Saudi Arabia	3	1624	105	0.0657 (0.0410–0.0956)	76	0.0018	P < .001
Cambodia	2	1040	184	0.1715 (0.1366–0.2094)	49	0.0006	P < .001
Thailand	13	11,525	1404	0.1431 (0.0752–0.2280)	99	0.0400	P < .001
Bangladesh	1	149	56	0.3758 (0.3021–0.4558)	–	–	–
Turkey	5	3045	497	0.1259 (0.0655–0.2022)	97	0.0134	P < .001
Malaysia	5	3032	704	0.3557 (0.1831–0.5502)	99	0.0490	P < .001
Vietnam	1	217	22	0.1014 (0.0679–0.1487)	–	–	–
Oman	1	436	157	0.3601 (0.3164–0.4062)	–	–	–
Syria	1	1469	341	0.2321 (0.2113–0.2544)	–	–	–
Yemen	9	4280	1735	0.4310 (0.2944–0.5731)	98	0.0467	P < .001
Tajikistan	1	594	497	0.8367 (0.8048–0.8642)	–	–	–
Iraq	3	1788	400	0.1590 (0.0659–0.2821)	96	0.0163	P < .001
Pakistan	6	1508	215	0.1066 (0.0434–0.1926)	95	0.0215	P < .001
Lebanon	1	249	212	0.8514 (0.8019–0.8902)	–	–	–
Afghanistan	1	500	97	0.1940 (0.1617–0.2309)	–	–	–
Total	131	106,225	20,230	0.2085 (0.1801–0.2383)	98	0.0426	P < .001
Income level							
High income	4	2060	262	0.1228 (0.0274–0.2719)	98	0.0369	P < .001
Upper middle income	32	19,311	2931	0.1831 (0.1299–0.2432)	98	0.0435	P < .001
Lower middle income	85	79,105	14,961	0.01977 (0.1677–0.2295)	98	0.0321	P < .001
Low income level	10	5749	2076	0.4096 (0.2824–0.5433)	98	0.0460	P < .001
Total	131	106,225	20,230	0.2085 (0.1801–0.2383)	98	0.0426	P < .001
Diagnostic method							
Direct smear & Concentration (Sedimentation)	50	60,743	12,070	0.2142 (0.1641–0.2689)	99	0.0519	P < .001
Direct smear	29	16,590	2387	0.1836 (0.1417–0.2295)	98	0.0231	P < .001
Concentration (Sedimentation)	12	5768	1477	0.2438 (0.1492–0.3529)	98	0.0434	P < .001
Concentration (Flotation)	1	285	1	0.0035 (0.0006–0.0196)	–	–	–
Direct smear & Concentration (Flotation & Sedimentation)	3	1680	285	0.1088 (0.0153–0.2682)	96	0.0322	P < .001
Direct smear & Concentration (Sedimentation) & Staining & PCR	2	1068	169	0.1676 (0.1095–0.2351)	87	0.0031	P < .001
Direct smear & Staining	9	103,638	659	0.1829 (0.1315–0.2405)	93	0.0106	P < .001
Direct smear & Culture	2	1114	239	0.2735 (0.0329–0.6298)	99	0.0690	P < .001
Direct smear & Concentration (Sedimentation) & Staining	11	8395	1614	0.2011 (0.1354–0.2760)	98	0.0217	P < .001
Direct smear & Concentration (Flotation) & Staining	1	712	247	0.3469 (0.3129–0.3826)	–	–	–
Direct smear & Concentration (Flotation)	2	1696	267	0.1766 (0.1065–0.2599)	92	0.0049	P < .001
Direct smear & Concentration (Sedimentation) & Culture	2	2023	260	0.1812 (0.0505–0.3681)	97	0.0228	P < .001
Staining	1	500	64	0.1280 (0.1015–0.1601)	–	–	–
Direct smear & Staining & PCR	1	249	212	0.8514 (0.8019–0.8902)	–	–	–
Direct smear & Concentration (Flotation & Sedimentation) & Staining	2	999	114	0.1670 (0.0154–0.4290)	98	0.0436	P < .001
Direct smear & Culture & PCR	3	765	165	0.2931 (0.0758–0.5782)	98	0.0645	P < .001
Total	131	106,225	20,230	0.2085 (0.1801–0.2383)	98	0.0426	P < .001
GBD geographies regions							
South Asia	56	21,220	3657	0.1645 (0.1333–0.1982)	96	0.0266	P < .001
North Africa and Middle East	44	67,295	13,657	0.2566 (0.2031–0.3140)	99	0.0456	P < .001
Southeast Asia	30	17,116	2636	0.2067 (0.1472–0.2733)	98	0.0460	P < .001
Central Asia	1	594	280	0.8367 (0.8048–0.8642)	–	–	–
Total	131	106,225	20,230	0.2085 (0.1801–0.2383)	98	0.0426	P < .001
Educational level							
Primary School	35	40,815	7753	0.2247 (0.1838–0.2684)	98	0.0225	P < .001
Elementary School	6	3519	1482	0.2527 (0.1131–0.4248)	99	0.0509	P < .001
Secondary school	2	4231	140	0.0583 (0.0085–0.1466)	94	0.0108	P < .001
School Children	88	57,660	10,855	0.1998 (0.1648–0.2372)	98	0.0458	P < .001
Total	131	106,225	20,230	0.2085 (0.1801–0.2383)	98	0.0426	P < .001

**Table 3:** Sub-group analysis based on type of protozoan parasites

Type of intestinal protozoan parasites	No studies	Sample size	Infected	Pooled prevalence (95% CI)	Heterogeneity		
					*I*^2^	τ^2^	*p*-value
***Blastocystis hominis***	46	64,110	3149	0.0794 (0.0519–0.1120)	98	0.0356	P < .001
***Entamoeba histolytica / dispar***	92	81,981	3450	0.0647 (0.0488–0.0825)	98	0.0265	P < .001
***Entamoeba coli***	55	66,772	2474	0.0510 (0.0340–0.0711)	98	0.0238	P < .001
***Cryptosporidium* spp.**	13	6979	541	0.0679 (0.0253–0.1286)	98	0.0349	P < .001
***Giardia duodenalis***	121	103,228	10,607	0.0824 (0.0702–0.0955)	97	0.0155	P < .001
***Endolimax nana***	22	12,828	340	0.0281 (0.0160–0.0434)	94	0.0086	P < .001
***Chilomastix mesnili***	9	8083	221	0.0100 (0.0012–0.0258)	98	0.0078	P < .001
***Iodamoeba butschlii***	19	9340	120	0.0137 (0.0051–0.0260)	87	0.0076	P < .001
***Balantidium coli***	1	184	1	0.0054 (0.0000–0.0232)	–	–	–
***Sarcocystis spp***	1	403	1	0.0025 (0.0000–0.0106)	–	–	–
***Isospora belli***	2	1646	7	0.0043 (0.0008–0.0100)	25	0.0002	P < .001
***Cyclospora spp***	5	2909	49	0.0161 (0.0078–0.0270)	62	0.0012	P < .001
***Entamoeba hartmanni***	6	4153	81	0.0259 (0.0060–0.0581)	95	0.0093	P < .001
***Trichomonas intestinalis***	1	2169	3	0.0014 (0.0002–0.0035)	–	–	–

### Quality assessment

We employed the Newcastle–Ottawa Scale to evaluate the quality of the study, as detailed in Supplementary Table 2 [15]. The scoring system was based on the following components and their respective score ranges:

- Selection (up to a maximum of 5 stars).
- Comparability (up to a maximum of 2 stars).
- Outcome (up to a maximum of 3 stars).

### Data synthesis and statistical analysis

Multiple statistical methods were employed to comprehensively analyze data concerning the prevalence of IPPs among schoolchildren in Asia. A 95% confidence interval (95% CI) was used to calculate the overall pooled prevalence. To estimate this pooled prevalence, a random-effects model with a Freeman-Tukey double arcsine transformation was utilized. To assess potential publication bias, Begg's rank test was applied, and publication bias was also evaluated using the Luis Furuya-Kanamori (LFK) index and the Doi plot [16]. An LFK index falling outside the ± 2, ± 2, and ± 1 range was considered significantly asymmetrical, slightly asymmetrical, and symmetrical (indicating the absence of publication bias), respectively.

Additionally, heterogeneity among the included studies was evaluated using Cochrane's Q test and the inconsistency index (*I*2 statistics), where *I*2 values of 0–25% were classified as low heterogeneity, 25–50% as moderate heterogeneity, and 50–75% as high heterogeneity. Statistical significance was defined as a p-value less than 0.05. All statistical analyses were conducted using the meta and metasens packages in R (version 3.6.1) [17]. This review was registered in PROSPERO (CRD42023481146).

## Results

### Characteristics of included studies

**Fig. 1:**
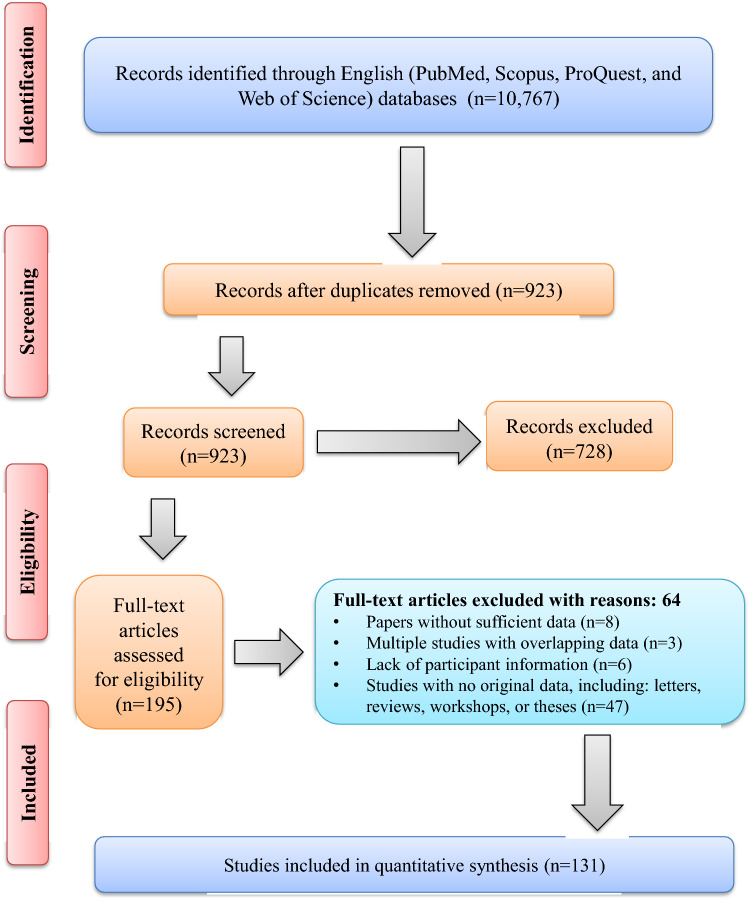
Flow diagram of the study design process

The current study involved a systematic search that resulted in the identification of 10,767 articles, out of which 195 full-text papers were selected for a detailed assessment to determine their eligibility. After our evaluations, we excluded eight studies due to lack of sufficient data, three studies that contained overlapping data, six studies that lacked essential participant details, and 47 studies that did not present original data, such as letters, reviews, workshops, and theses. Finally, 131 papers met the critical appraisal criteria for inclusion in the meta-analysis (Fig. 1).

The prevalence of IPPs among schoolchildren has been reported in thirty-two Asian countries. The largest number of reports were related to Nepal (35 studies), followed by Iran (15 studies) (Table 2).

**Fig. 2:**
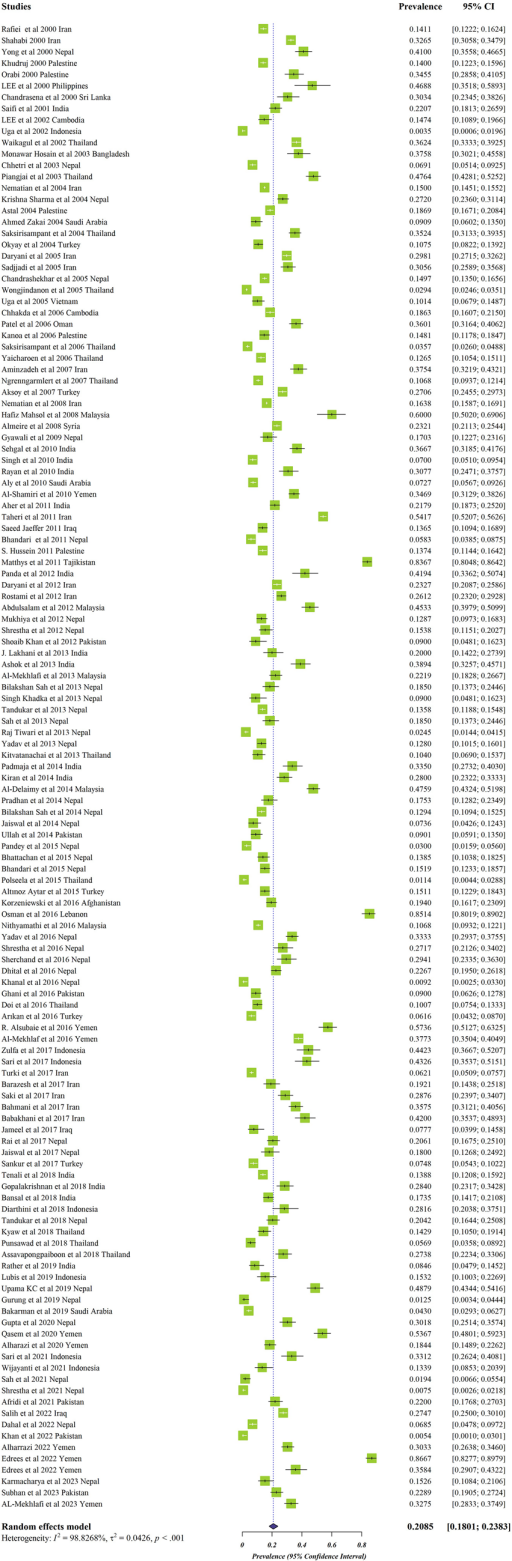
Forest plots for random-effects meta-analysis of intestinal protozoan parasites among Asian schoolchildren (the box indicate the effect size of the studies (prevalance) and the whiskers indicate its confidence interval for corresponding effect size. There is no specific difference between white and black bars, only studies with a very narrow confidence interval are shown in white. In the case of diamonds, their size indicates the size of the effect, and their length indicate confidence intervals)

The estimated pooled prevalence of IPPs among Asian schoolchildren was 0.208 (95% CI = 0.180–0.238) (Fig. 2).

The studies included in this review employed parasitology techniques comprising microscopic methods (concentration with and without flotation or sedimentation, and other direct smear techniques), culture method, molecular approach (conventional PCR and real-time PCR), and staining methods (Lugol's iodine, trichrome, and Ziehl–Neelsen) (Table 2).

**Fig. 3:**
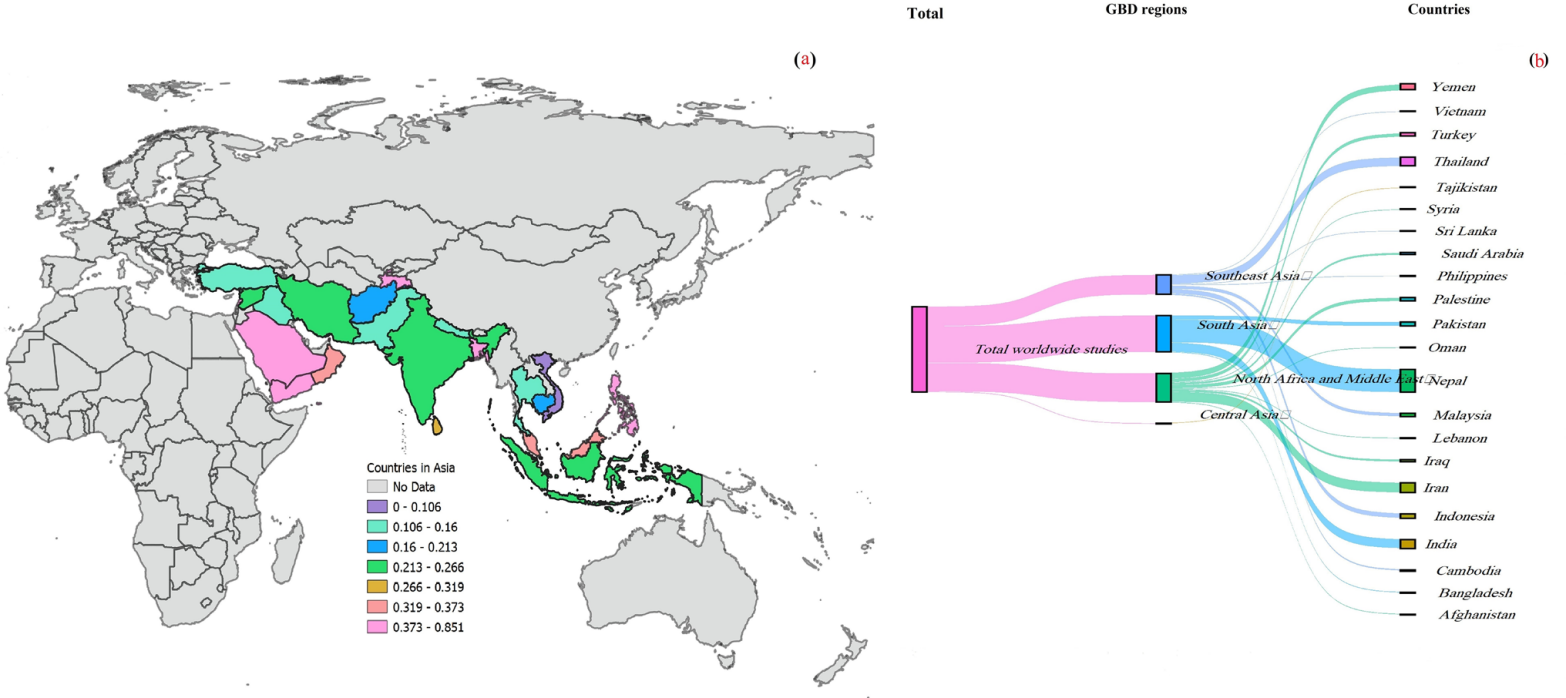
(**a**) Geographical region where the study was published, (**b**) distribution of intestinal protozoan parasites among Asian schoolchildren per GDB Geography

Based on our included studies, we designed a map using QGIS3 software (https://qgis.org/en/site/) to display the prevalence of IPPs among schoolchildren in different regions of Asia (Fig. 3).

### Pooled prevalence based on the type of intestinal protozoan parasites, source of samples, gender, and diagnostic techniques

The pooled prevalence of IPPs among Asian schoolchildren was estimated as follows; 0.082 (95% CI = 0.070–0.095) for *G. duodenalis* with heterogeneity (*I*^*2*^ = 97; τ^2^ = 0.015; p < 0.001), 0.079 (95% CI = 0.051–0.112) for *Blastocystis hominis* with heterogeneity (*I*^*2*^ = 98; τ^2^ = 0.035; p < 0.001), 0.067 (95% CI = 0.025–0.128) for *Cryptosporidium* spp. with heterogeneity (*I*^*2*^ = 98; τ^2^ = 0.034; p < 0.001), 0.064 (95% CI = 0.048–0.082) for *E. histolytica/dispar* with heterogeneity (*I*^*2*^ = 98; τ^2^ = 0.026; p < 0.001), 0.051 (95% CI = 0.034–0.071) for *E. coli* with heterogeneity (*I*^*2*^ = 98; τ^2^ = 0.023; p < 0.001), 0.028 (95% CI = 0.016–0.043) for *Endolimax nana* with heterogeneity (*I*^*2*^ = 94; τ^2^ = 0.008; p < 0.001), 0.025 (95% CI = 0.006–0.058) for *E. hartmanni* with heterogeneity (*I*^*2*^ = 95; τ^2^ = 0.009; p < 0.001), 0.016 (95% CI = 0.007–0.027) for *Cyclospora* cayetanensis with heterogeneity (*I*^*2*^ = 62; τ^2^ = 0.001; p < 0.001), 0.013 (95% CI = 0.005–0.026) for *Iodamoeba buetschlii* with heterogeneity (*I*^*2*^ = 87; τ^2^ = 0.007; p < 0.001), 0.010 (95% CI = 0.001–0.025) for *Chilomastix mesnili* with heterogeneity (*I*^*2*^ = 98; τ^2^ = 0.007; p < 0.001), 0.005 (95% CI = 0–0.023) for *Balantidium coli*, 0.004 (95% CI = 0.0008–0.010) for *Cystoisospora belli* with heterogeneity (*I*^*2*^ = 25; τ^2^ = 0.0002; p < 0.001), and 0.002 (95% CI = 0–0.010) for *Sarcocystis* spp*.* (Table 3).

The highest pooled prevalence estimated based on the source of samples was 0.208 (95% CI = 0.180–0.237) with heterogeneity (*I*^*2*^ = 98; τ^2^ = 0.039; p < 0.001) for stool (Table 2).

The results of this study show that the male/female ratio is approximately equal for both sexes (OR: 1.019, 95% CI, 0.842–1.234) (Supplementary Fig. 1).

Based on the diagnostic techniques, the highest pooled prevalence (0.851, 95% CI = 0.801–0.890) was related to studies that employed a combined direct smear, staining, and PCR methods (Table 2).

### Pooled prevalence based on GBD regions, country, socio-economic status, and educational level

According to different GBD regions, the pooled prevalence ranged from 0.836% to 0.164%, including 0.836 (95% CI = 0.804–0.864) for Central Asia, 0.256 (95% CI = 0.203–0.314) for North Africa and Middle East with heterogeneity (*I*^*2*^ = 99; τ^2^ = 0.045; p < 0.001), 0.206 (95% CI = 0.147–0.273) for Southeast Asia with heterogeneity (*I*^*2*^ = 98; τ^2^ = 0.046; p < 0.001), and 0.164 (95% CI = 0.133–0.198) for South Asia with heterogeneity (*I*^*2*^ = 96; τ^2^ = 0.026; p < 0.001) (Table 2).

At the country level, the highest pooled prevalence was observed for Lebanon (0.851, 95% CI = 0.801–0.890) and Tajikistan (0.836, 95% CI = 0.804–0.864), both with one study (Table 2).

The pooled prevalence estimated based on income level ranged from 0.122 to 0.409, with the highest rate related to the low-income group (0.409, 95% CI = 0.282–0.543) with heterogeneity (*I*^*2*^ = 98; τ^2^ = 0.046; p < 0.001) (Table 2). Furthermore, based on educational level, the IPPs were most prevalent in the elementary school group (0.252, 95% CI = 0.113–0.424) with heterogeneity (*I*^*2*^ = 99; τ^2^ = 0.050; p < 0.001) (Table 2).

### Pooled prevalence based on climate variables

Our analyses indicated that IPPs were most prevalent in schoolchildren in regions with annual rainfall of < 400 mm (0.260, 95% CI = 0.203–0.322) and humidity of < 30 (0.324 (95% CI = 0.254–0.398) with heterogeneities (*I*^*2*^ = 99; τ^2^ = 0.039; p < 0.001) and (*I*^*2*^ = 99; τ^2^ = 0.036; p < 0.001), respectively (Table 2).

Moreover, regions with an average temperature of > 20 °C represent the highest rate of prevalence (0.206, 95% CI = 0.166–0.250) with heterogeneity (*I*^*2*^ = 98; τ^2^ = 0.042; p < 0.001) (Table 2).

Based on climate, we found that regions with tropical rainforest climate had the highest pooled prevalence (0.291, 95% CI = 0.192–0.401) with heterogeneity (*I*^*2*^ = 98; τ^2^ = 0.046; p < 0.001) (Table 2).

### Meta-regression

**Fig. 4:**
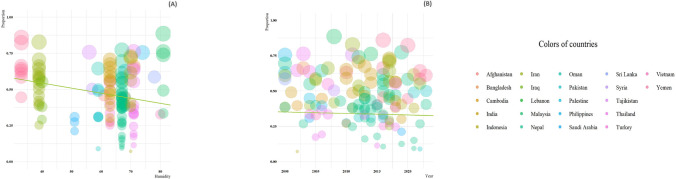
A meta-regression graph for the prevalnce of intestinal protozoan parasites among Asian schoolchildren based on humidity (**A**), and year of publication (**B**). The pink line is the regression line, which was plotted based on the intercept and the slope of the regession model. The different colour bubbles represent the countries under study and their sizes indicates the effect size of each study

Heterogeneity was observed for humidity and year of publication. Accordingly, the test showed a statistically significance result for humidity (slop = 0.0009, p < 0.0061) and year of publication (slop = 0.0044, p < 0.0065) for all studies included in the current review (Fig. 4A, B).

### Publication bias and sensitivity analysis

**Fig. 5:**
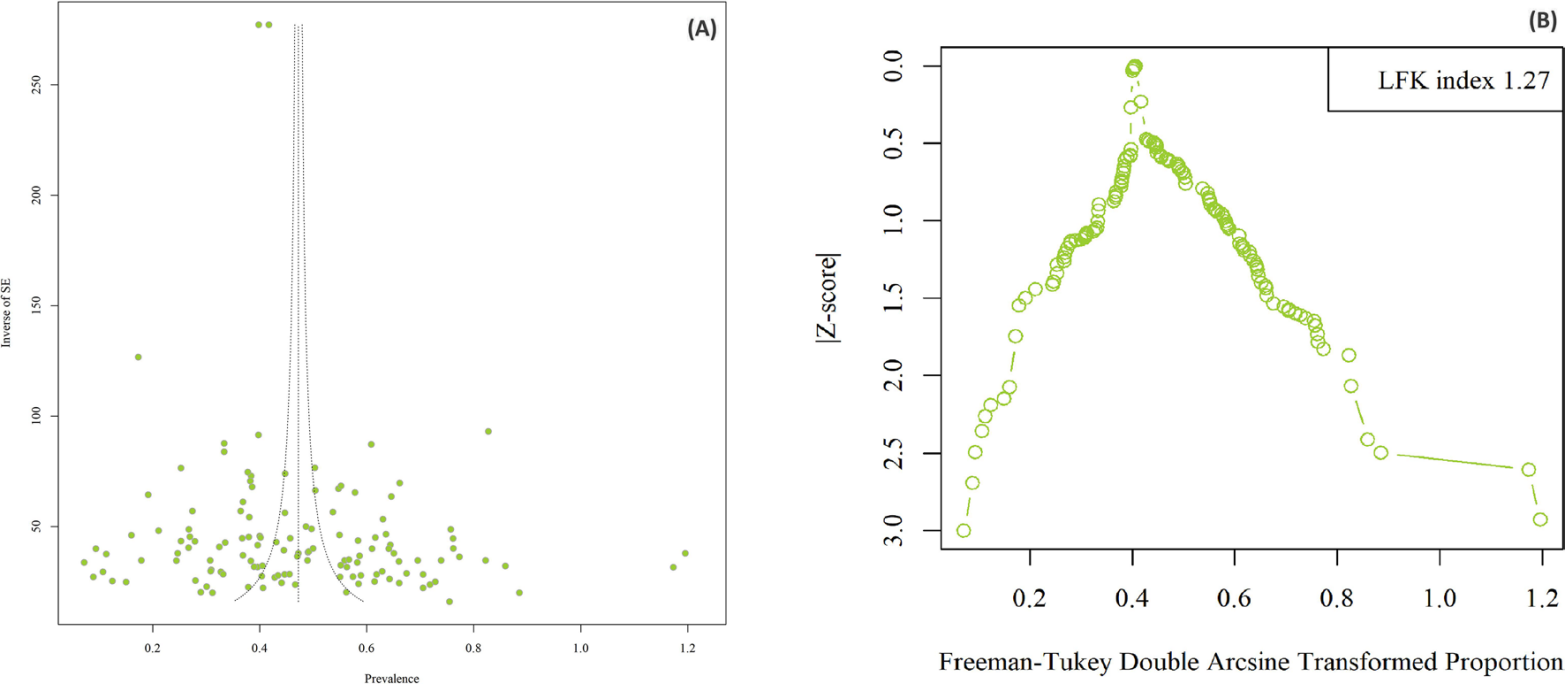
Egger's funnel plot to access publication bias in studies evaluating of intestinal protozoan parasites among Asian schoolchildren (**A**). Doi plot: A Luis Furuya-Kanamori (LFK) index 1.27 indicates minor asymmetry (**B**)

There was no significant publication bias according to Egger’s test (t = 1.10, p = 0.271). Based on the Doi plot test, there was a minor asymmetry (LFK index: 1.27) (Fig. 5A, B). The sensitivity analysis results indicated that the impact of each study on the overall estimates of the current meta-analysis was not statistically significant (Supplementary Fig. 2).

A Sankey plot is designed using R software (version 3.6.1) to represent the taxa of intestinal protozoan parasites studies in Asian schoolchildren per taxonomic order, family, and genus (Supplementary Fig. 3).

### Quality assessment

Following the quality assessment, it was revealed that among the 131 studies, 95 were classified as high quality (score of 7–9 points), and 36 were categorized as moderate quality (score of 4–6 points) (Supplementary Table 2).

## Discussion

Protozoan parasites play a substantial role in the global public health challenge, particularly affecting developing regions where these infections are prevalent and pose a significant threat to community well-being, with children at heightened risk [1, 18].

To the best of our knowledge, this is the first systematic review and meta-analysis assessing the IPP prevalence among schoolchildren in Asia across the continent. In this study, the prevalence of IPPs among schoolchildren was determined to be relatively high (0.208%), which may be attributable to inadequate hygiene practices among these populations.

Although public health measures are generally stricter in developed countries compared to developing ones, minority groups, institutionalized individuals, and the immunocompromised remain at very high risk. This risk can extend to the broader population, making these groups a public health priority. *Giardia duodenalis*, *Cryptosporidium* spp., and *Entamoeba* spp. are the most frequently reported protozoa linked to enteric infections, and are primarily associated with outbreaks originating from food and water sources. Other protozoa, such as *C. cayetanensis*, *B. coli*, *C. belli*, and *Blastocystis* spp., are becoming significant causes of illness, particularly affecting travelers to developing regions, immunocompromised individuals, and young children. [1]. The public health sectors recommendations can be affected by the severity and pathogenesis of infections caused by protozoan parasites. The serious clinical symptoms, such as dehydration, malnutrition, or organ damage may lead to public health authorities prioritizing approaches for prevention, detection, and treatment. For instance, in regions with a high prevalence of water- and foodborne protozoan parasites like *Giardia* and *Cryptosporidium*, and outbreaks are possible, recommendations may prioritize measures such as enhancing food safety, improving water treatment and sanitation, and educating the public on hygiene practices [19–21].

Our results follow a prior study conducted among Asian children, which documented that *G. duodenalis* (15.1%) was the predominant intestinal parasite [22].

*G. duodenalis* is known to infect around 200 million people globally, with a higher prevalence rate among schoolchildren and in daycare centers [23]. The primary mode of transmission for *G. duodenalis* is through the fecal–oral route [24], and the primary sources of transmission include drinking water, food, and vegetables contaminated with cysts of the parasite [25, 26].

In a recent meta-analysis, the study revealed that the factors associated with an increased risk of giardiasis include being exposed to sewage or wastewater, consuming untreated drinking water, and engaging in recreational activities in water. Remarkably, the study also found that having contact with pets was a significant risk factor for giardiasis, particularly in children. Moreover, traveling to foreign countries was identified as another risk factor, especially in industrialized nations [22]. In children under the age of five, giardiasis can result in the development of severe, acute diarrhea, and several research studies have put evidence suggesting that chronic giardiasis, if left untreated or recurrent, may have lasting consequences in terms of growth retardation, impacting the long-term physical development of affected individuals [27]. This highlights the significance of addressing and managing giardiasis, especially in young children, to mitigate potential health and growth-related issues.

Our country-based analysis revealed that Lebanon and Tajikistan represent the highest prevalence rates of IPPs. However, we need to interpret it cautiously due to the low number of studies related to these two countries, which may cause bias in the results towards a higher prevalence rate. In Lebanon, many households lack adequate sanitation systems, leading to fecal contamination through ground seepage.

Lebanon faced an issue regarding wastewater management, which resulted in large-scale water pollution. According to research, nearly 74% of samples collected from rivers, which are the primary source of irrigation water in the country, surpassed the microbiological acceptability standards for this purpose. Moreover, in this country, the water sources are poorly managed, and freshwater is scarce, leading farmers to rely highly on untreated water sources [28].

In Tajikistan, it is estimated that approximately half of the rural households rely on untreated water sources for drinking, and this failure to meet the drinking water and poor sanitation standards in the country can partly be attributed to the contaminated water supply, which is associated with transmission of both waterborne and foodborne parasitic protozoa [29, 30].

Our findings based on different GBD regions found that Central Asia accounted for the highest prevalence rate of IPPs, which was in parallel with our country-based results. In this part of Asia, in Kyrgyzstan, a survey on children between the ages of 6 and 15 revealed that the overall prevalence of IPPs was found to be relatively high (41%) [31].

In Central Asian countries, the limited availability of high-quality drinking water is one of several issues related to public health. The aging and poor condition of water pipeline networks lead people to resort to alternative, often untreated, water sources. Additional factors also contribute significantly to the problem, including contamination of the water supply sources discharges from industrial and agricultural activities [32].

The infection rate is directly linked to factors such as sanitation, proper disposal of feces with good hygiene practices, access to safe drinking water, and other related factors [33, 34]. In regions with poor sanitary settings, public tap or standpipes as a source of drinking water supply emerged as a protective factor against IPPs, particularly concerning giardiasis [35].

Multiple reports have consistently highlighted the increased occurrence of IPPs in poor communities residing in countries with low to lower-middle income status, with a particular focus on various Asian nations. As anticipated, our review showed that the most significant incidence of IPPs in schoolchildren occurred in countries characterized by low and lower-middle income levels [36, 37].

Our review highlighted that regions with a tropical rainforest climate had the highest incidence of IPPs among schoolchildren in Asia, underscoring the significance of climate conditions as essential factors that affect the prevalence of IPPs in this context.

Parasitic intestinal infections are prevalent in tropical and subtropical regions, particularly in areas like Sub-Saharan Africa, Latin America, China, and East Asia. The warm and humid climate in these regions creates favorable conditions for the transmission and distribution of parasites, contributing to the high prevalence of these infections [6]. Furthermore, these regions experience significant population growth coupled with elevated poverty rates, which further escalates the risk of parasite transmission [38–40].

In the current study, the highest prevalence of IPPs was related to primary and elementary school-aged children, which might be attributed to their weaker immune systems, increased contact with soil and other contaminated materials, and their limited adherence to health standards [41].

Based on our analysis of various methods, the highest prevalence was related to the studies that employed a combination of direct smear, staining, and PCR techniques that are regarded as an approach for the qualitative diagnosis of intestinal protozoan parasites. PCR offers a higher detection sensitivity than light microscopy, making it particularly valuable for identifying a low number of parasites in stool samples [42]. Nevertheless, using a combination of microscopy with immunoassay or molecular methods has resulted in a notable improvement in both sensitivity and specificity over the last two decades [43].

## Limitations

This study has faced particular limitations that should be noted. Firstly, there were limitations in the number of studies available for specific subgroups of Asian schoolchildren. In some cases, only one article addressed the prevalence of protozoan parasites for certain types of parasites. Secondly, our analyses might have been influenced by publication bias, stemming from the absence of or a limited number of studies available from specific geographical regions. Lastly, some of the studies in our analyses exhibited small-study effects, which can be attributed to factors such as limited sample size and the absence of a susceptible highly sensitive diagnostic technique. Despite these limitations, it's essential to acknowledge that this study offers the most comprehensive insights into the prevalence of intestinal protozoan parasites among Asian schoolchildren.

## Conclusion

The final remarks summarize the findings of the examination and meta-analysis, affirming the prevailing risk factors like climate and socioeconomic aspects in certain Asian countries. This underscores the ongoing risk of protozoan infections to children, along with the necessity to monitor the protozoan infection trends/patterns. It is imperative to update public health recommendations for surveillance of protozoan infections particularly given the emerging links between certain protozoan pathogenesis and other chronic diseases, and infections.

## Author contributors

MB, AVE, and PK contributed to the study design. ZM, ON, AKS, and MP searched for primary publications, screened, and appraised primary studies. ZM and AA extracted the data. MB, ON, and AVE contributed to the methodology. MO made a contribution to data analysis and interpretation. AVE, MB, and PK wrote the study manuscript. MB, AVE, and PK reviewed and edited the manuscript. All authors read the manuscript and participated in preparing the final version.

## Supplementary Information

Below is the link to the electronic supplementary material.

Supplementary file1 (PDF 464 KB)

Supplementary file2 (PDF 2327 KB)

Supplementary file3 (PDF 1130 KB)

Supplementary file4 (DOCX 19 KB)

Supplementary file5 (DOCX 38 KB)

## Data Availability

The datasets used and/or analyzed during the current study are included in the manuscript.
