# Supplementary material for: Prevalence of intestinal protozoan parasites among Asian schoolchildren: a systematic review and meta-analysis
Source: Infection. 2024 Jul 9;52(6):2097–133. doi: 10.1007/s15010-024-02339-1 (PMC11621188; doi:10.1007/s15010-024-02339-1)
Supplement: Supplementary file 1 — Supplementary file1 (PDF 464 KB) [file 15010_2024_2339_MOESM1_ESM.pdf]

Studies	OR	95% CI
Rafiei et al Iran 2000	0.8784	[0.6306; 1.2236]
Orabi Palestine 2000	0.7806	[0.4473; 1.3622]
Saifi et al India 2001	1.2910	[0.7287; 2.2870]
LEE et al Cambodia 2002	0.8468	[0.4212; 1.7023]
Uga et al Vietnam 2005	0.7243	[0.2905; 1.8056]
Matthys et al Tajikistan 2011	0.9119	[0.5896; 1.4104]
S. Hussein Palestine 2011	1.0925	[0.7127; 1.6747]
Saeed Jaeffer Iraq 2011	0.7720	[0.4660; 1.2789]
Taheri et al Iran 2011	0.9737	[0.8219; 1.1536]
Panda et al India 2012	0.7931	[0.3875; 1.6231]
Rostami et al Iran 2012	1.0580	[0.7657; 1.4617]
Al-Mekhlafi et al Malaysia 2013	1.3037	[0.7997; 2.1254]
Sah et al Nepal 2013	0.9927	[0.4861; 2.0275]
Yadav et al Nepal 2013	0.2832	[0.1631; 0.4918]
Sherchand et al Nepal 2016	0.5884	[0.3120; 1.1097]
Osman et al Lebanon 2016	1.3211	[0.6541; 2.6681]
R. Alsubaie et al Yemen 2016	3.0809	[1.8196; 5.2164]
Al-Mekhlaf et al Yemen 2016	1.0052	[0.7872; 1.2837]
Sankur et al Turkey 2017	1.0504	[0.5273; 2.0925]
Saki et al Iran 2017	1.5213	[0.9085; 2.5475]
Babakhani et al Iran 2017	2.3021	[1.2956; 4.0905]
Alharrazi Yemen 2022	1.1196	[0.7543; 1.6619]
Subhan et al Pakistan 2023	1.0244	[0.6415; 1.6358]
**Common effect model**	**1.0121**	**[0.9283; 1.1035]**
**Random effects model**	**1.0195**	**[0.8421; 1.2343]**

Heterogeneity: $I^2 = 61.8660\%$, $\tau^2 = 0.1476$, $p < .001$

Odds Ratio

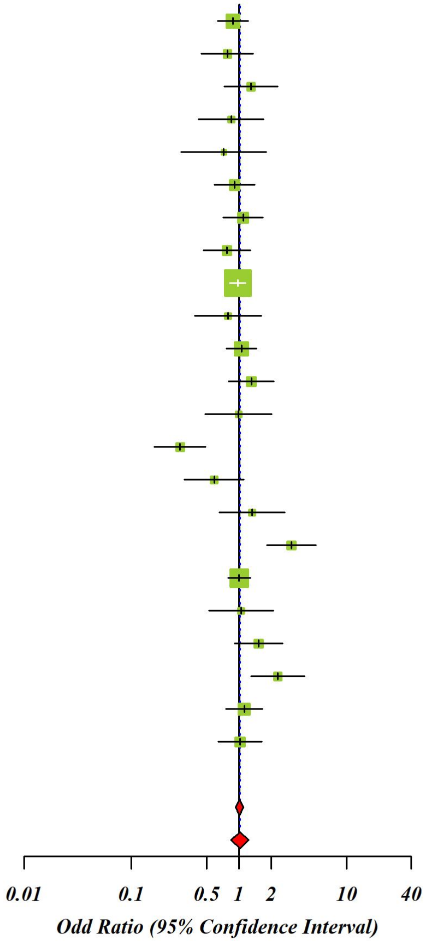

Supplementary Figure 1. Forest plot of odds ratios for gender and intestinal protozoan parasites among Asian schoolchildren.
