# Supplementary material for: Prevalence of intestinal protozoan parasites among Asian schoolchildren: a systematic review and meta-analysis
Source: Infection. 2024 Jul 9;52(6):2097–133. doi: 10.1007/s15010-024-02339-1 (PMC11621188; doi:10.1007/s15010-024-02339-1)
Supplement: Supplementary file 2 — Supplementary file2 (PDF 2327 KB) [file 15010_2024_2339_MOESM2_ESM.pdf]

Studies		Prevalence	95% CI
Omitting Khudruj 2000 Palestine		0.2065	[0.1790; 0.2354]
Omitting LEE et al 2000 Philippines		0.2043	[0.1771; 0.2330]
Omitting Rafiei et al 2000 Iran		0.2065	[0.1790; 0.2354]
Omitting Orabi 2000 Palestine		0.2050	[0.1776; 0.2338]
Omitting Yong et al 2000 Nepal		0.2045	[0.1772; 0.2333]
Omitting Shahabi 2000 Iran		0.2051	[0.1777; 0.2339]
Omitting Chandrasena et al 2000 Sri Lanka		0.2053	[0.1778; 0.2341]
Omitting Saifi et al 2001 India		0.2058	[0.1783; 0.2348]
Omitting Uga et al 2002 Indonesia		0.2084	[0.1812; 0.2370]
Omitting LEE et al 2002 Cambodia		0.2064	[0.1789; 0.2354]
Omitting Waikagul et al 2002 Thailand		0.2048	[0.1775; 0.2336]
Omitting Monawar Hosain et al 2003 Bangladesh		0.2048	[0.1774; 0.2336]
Omitting Piangjai et al 2003 Thailand		0.2041	[0.1769; 0.2327]
Omitting Chhetri et al 2003 Nepal		0.2072	[0.1798; 0.2361]
Omitting Ahmed Zakai 2004 Saudi Arabia		0.2070	[0.1795; 0.2359]
Omitting Nematian et al 2004 Iran		0.2064	[0.1789; 0.2353]
Omitting Astal 2004 Palestine		0.2061	[0.1786; 0.2350]
Omitting Okyay et al 2004 Turkey		0.2068	[0.1793; 0.2357]
Omitting Krishna Sharma et al 2004 Nepal		0.2055	[0.1780; 0.2343]
Omitting Saksirisampant et al 2004 Thailand		0.2049	[0.1775; 0.2337]
Omitting Daryani et al 2005 Iran		0.2053	[0.1778; 0.2341]
Omitting Chandrashekhar et al 2005 Nepal		0.2064	[0.1789; 0.2353]
Omitting Sadjjadi et al 2005 Iran		0.2052	[0.1778; 0.2341]
Omitting Uga et al 2005 Vietnam		0.2068	[0.1794; 0.2358]
Omitting Wongjindanon et al 2005 Thailand		0.2078	[0.1805; 0.2366]
Omitting Chhakda et al 2006 Cambodia		0.2061	[0.1786; 0.2350]
Omitting Kanoa et al 2006 Palestine		0.2064	[0.1789; 0.2354]
Omitting Patel et al 2006 Oman		0.2049	[0.1775; 0.2337]
Omitting Saksirisampant et al 2006 Thailand		0.2077	[0.1803; 0.2365]
Omitting Yaicharoen et al 2006 Thailand		0.2066	[0.1791; 0.2355]
Omitting Aksoy et al 2007 Turkey		0.2055	[0.1780; 0.2343]
Omitting Aminzadeh et al 2007 Iran		0.2048	[0.1774; 0.2335]
Omitting Ngrenngarmlert et al 2007 Thailand		0.2068	[0.1793; 0.2357]
Omitting Hafiz Mahsol et al 2008 Malaysia		0.2035	[0.1765; 0.2318]
Omitting Nematian et al 2008 Iran		0.2063	[0.1788; 0.2352]
Omitting Almeire et al 2008 Syria		0.2057	[0.1782; 0.2347]
Omitting Gyawali et al 2009 Nepal		0.2062	[0.1787; 0.2352]
Omitting Al-Shamiri et al 2010 Yemen		0.2049	[0.1776; 0.2337]
Omitting Aly et al 2010 Saudi Arabia		0.2072	[0.1797; 0.2361]
Omitting Sehgal et al 2010 India		0.2048	[0.1775; 0.2336]
Omitting Singh et al 2010 India		0.2072	[0.1798; 0.2361]
Omitting Rayan et al 2010 India		0.2052	[0.1778; 0.2341]
Omitting Matthys et al 2011 Tajikistan		0.2041	[0.1769; 0.2327]
Omitting S. Hussein 2011 Palestine		0.2065	[0.1790; 0.2355]
Omitting Saeed Jaeffer 2011 Iraq		0.2065	[0.1790; 0.2355]
Omitting Taheri et al 2011 Iran		0.2037	[0.1766; 0.2321]
Omitting Bhandari et al 2011 Nepal		0.2074	[0.1799; 0.2362]
Omitting Aher et al 2011 India		0.2059	[0.1784; 0.2348]
Omitting Abdulsalam et al 2012 Malaysia		0.2043	[0.1770; 0.2329]
Omitting Daryani et al 2012 Iran		0.2057	[0.1782; 0.2347]
Omitting Mukhiya et al 2012 Nepal		0.2066	[0.1791; 0.2355]
Omitting Panda et al 2012 India		0.2045	[0.1772; 0.2332]
Omitting Rostami et al 2012 Iran		0.2055	[0.1781; 0.2344]
Omitting Shrestha et al 2012 Nepal		0.2064	[0.1789; 0.2353]
Omitting Shoaib Khan et al 2012 Pakistan		0.2069	[0.1794; 0.2358]
Omitting Al-Mekhlafi et al 2013 Malaysia		0.2058	[0.1783; 0.2347]
Omitting Bilakshan Sah et al 2013 Nepal		0.2061	[0.1786; 0.2350]
Omitting Singh Khadka et al 2013 Nepal		0.2069	[0.1794; 0.2358]
Omitting J. Lakhani et al 2013 India		0.2060	[0.1785; 0.2349]
Omitting Kitvatanachai et al 2013 Thailand		0.2068	[0.1793; 0.2357]
Omitting Ashok et al 2013 India		0.2047	[0.1774; 0.2334]
Omitting Tandukar et al 2013 Nepal		0.2065	[0.1790; 0.2355]
Omitting Sah et al 2013 Nepal		0.2061	[0.1786; 0.2350]
Omitting Raj Tiwari et al 2013 Nepal		0.2079	[0.1806; 0.2366]
Omitting Yadav et al 2013 Nepal		0.2066	[0.1791; 0.2355]
Omitting Ullah et al 2014 Pakistan		0.2070	[0.1795; 0.2359]
Omitting Pradhan et al 2014 Nepal		0.2062	[0.1787; 0.2351]
Omitting Padmaja et al 2014 India		0.2050	[0.1776; 0.2339]
Omitting Kiran et al 2014 India		0.2054	[0.1779; 0.2343]
Omitting Bilakshan Sah et al 2014 Nepal		0.2066	[0.1791; 0.2355]
Omitting Jaiswal et al 2014 Nepal		0.2071	[0.1797; 0.2360]
Omitting Al-Delaimy et al 2014 Malaysia		0.2041	[0.1769; 0.2327]
Omitting Pandey et al 2015 Nepal		0.2078	[0.1804; 0.2365]
Omitting Polseela et al 2015 Thailand		0.2082	[0.1809; 0.2369]
Omitting Altınoz Aytar et al 2015 Turkey		0.2064	[0.1789; 0.2353]
Omitting Bhattachan et al 2015 Nepal		0.2065	[0.1790; 0.2354]
Omitting Bhandari et al 2015 Nepal		0.2064	[0.1789; 0.2353]
Omitting Yadav et al 2016 Nepal		0.2050	[0.1776; 0.2339]
Omitting Shrestha et al 2016 Nepal		0.2055	[0.1780; 0.2344]
Omitting Sherchand et al 2016 Nepal		0.2053	[0.1779; 0.2342]
Omitting Dhital et al 2016 Nepal		0.2058	[0.1783; 0.2347]
Omitting Doi et al 2016 Thailand		0.2069	[0.1794; 0.2358]
Omitting Osman et al 2016 Lebanon		0.2016	[0.1756; 0.2289]
Omitting Arıkan et al 2016 Turkey		0.2073	[0.1799; 0.2362]
Omitting Nithyamathi et al 2016 Malaysia		0.2068	[0.1793; 0.2357]
Omitting Korzeniewski et al 2016 Afghanistan		0.2060	[0.1785; 0.2350]
Omitting Khanal et al 2016 Nepal		0.2082	[0.1809; 0.2369]
Omitting R. Alsubaie et al 2016 Yemen		0.2035	[0.1765; 0.2319]
Omitting Al-Mekhlaf et al 2016 Yemen		0.2047	[0.1774; 0.2335]
Omitting Ghani et al 2016 Pakistan		0.2070	[0.1795; 0.2359]
Omitting Zulfa et al 2017 Indonesia		0.2044	[0.1771; 0.2330]
Omitting Turki et al 2017 Iran		0.2073	[0.1799; 0.2362]
Omitting Sankur et al 2017 Turkey		0.2072	[0.1797; 0.2360]
Omitting Barazesh et al 2017 Iran		0.2061	[0.1785; 0.2350]
Omitting Sari et al 2017 Indonesia		0.2044	[0.1772; 0.2331]
Omitting Saki et al 2017 Iran		0.2054	[0.1779; 0.2342]
Omitting Rai et al 2017 Nepal		0.2059	[0.1784; 0.2349]
Omitting Bahmani et al 2017 Iran		0.2049	[0.1775; 0.2337]
Omitting Jameel et al 2017 Iraq		0.2071	[0.1796; 0.2359]
Omitting Babakhani et al 2017 Iran		0.2045	[0.1772; 0.2332]
Omitting Jaiswal et al 2017 Nepal		0.2061	[0.1786; 0.2351]
Omitting Tenali et al 2018 India		0.2065	[0.1790; 0.2354]
Omitting Kyaw et al 2018 Thailand		0.2065	[0.1789; 0.2354]
Omitting Tandukar et al 2018 Nepal		0.2060	[0.1785; 0.2349]
Omitting Punsawad et al 2018 Thailand		0.2074	[0.1799; 0.2362]
Omitting Gopalakrishnan et al 2018 India		0.2054	[0.1779; 0.2343]
Omitting Diarthini et al 2018 Indonesia		0.2054	[0.1780; 0.2343]
Omitting Assavapongpaiboon et al 2018 Thailand		0.2055	[0.1780; 0.2343]
Omitting Bansal et al 2018 India		0.2062	[0.1787; 0.2351]
Omitting Upama KC et al 2019 Nepal		0.2041	[0.1769; 0.2326]
Omitting Rather et al 2019 India		0.2070	[0.1795; 0.2359]
Omitting Gurung et al 2019 Nepal		0.2081	[0.1808; 0.2368]
Omitting Bakarman et al 2019 Saudi Arabia		0.2076	[0.1802; 0.2364]
Omitting Lubis et al 2019 Indonesia		0.2064	[0.1788; 0.2353]
Omitting Qasem et al 2020 Yemen		0.2038	[0.1767; 0.2322]
Omitting Gupta et al 2020 Nepal		0.2053	[0.1778; 0.2341]
Omitting Alharazi et al 2020 Yemen		0.2061	[0.1786; 0.2350]
Omitting Afridi et al 2021 Pakistan		0.2058	[0.1783; 0.2348]
Omitting Sari et al 2021 Indonesia		0.2051	[0.1777; 0.2339]
Omitting Sah et al 2021 Nepal		0.2079	[0.1806; 0.2367]
Omitting Shrestha et al 2021 Nepal		0.2083	[0.1811; 0.2369]
Omitting Wijayanti et al 2021 Indonesia		0.2065	[0.1790; 0.2354]
Omitting Alharrazi 2022 Yemen		0.2052	[0.1778; 0.2341]
Omitting Dahal et al 2022 Nepal		0.2072	[0.1798; 0.2361]
Omitting Edrees et al 2022 Yemen		0.2014	[0.1755; 0.2286]
Omitting Edrees et al 2022 Yemen		0.2049	[0.1775; 0.2337]
Omitting Khan et al 2022 Pakistan		0.2083	[0.1811; 0.2369]
Omitting Salih et al 2022 Iraq		0.2054	[0.1780; 0.2343]
Omitting AL-Mekhlafi et al 2023 Yemen		0.2051	[0.1777; 0.2339]
Omitting Karmacharya et al 2023 Nepal		0.2064	[0.1789; 0.2353]
Omitting Subhan et al 2023 Pakistan		0.2058	[0.1783; 0.2347]
**Random effects model**		**0.2059**	**[0.1786; 0.2346]**

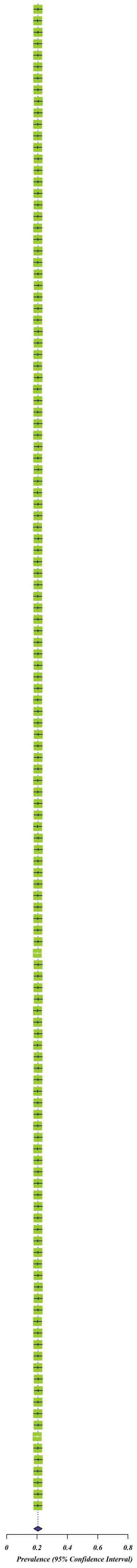

Supplementary Figure 2. Sensitivity analysis for assessing the effect of each primary study on the total estimates.
