# Supplementary material for: Prevalence of intestinal protozoan parasites among Asian schoolchildren: a systematic review and meta-analysis
Source: Infection. 2024 Jul 9;52(6):2097–133. doi: 10.1007/s15010-024-02339-1 (PMC11621188; doi:10.1007/s15010-024-02339-1)
Supplement: Supplementary file 3 — Supplementary file3 (PDF 1130 KB) [file 15010_2024_2339_MOESM3_ESM.pdf]

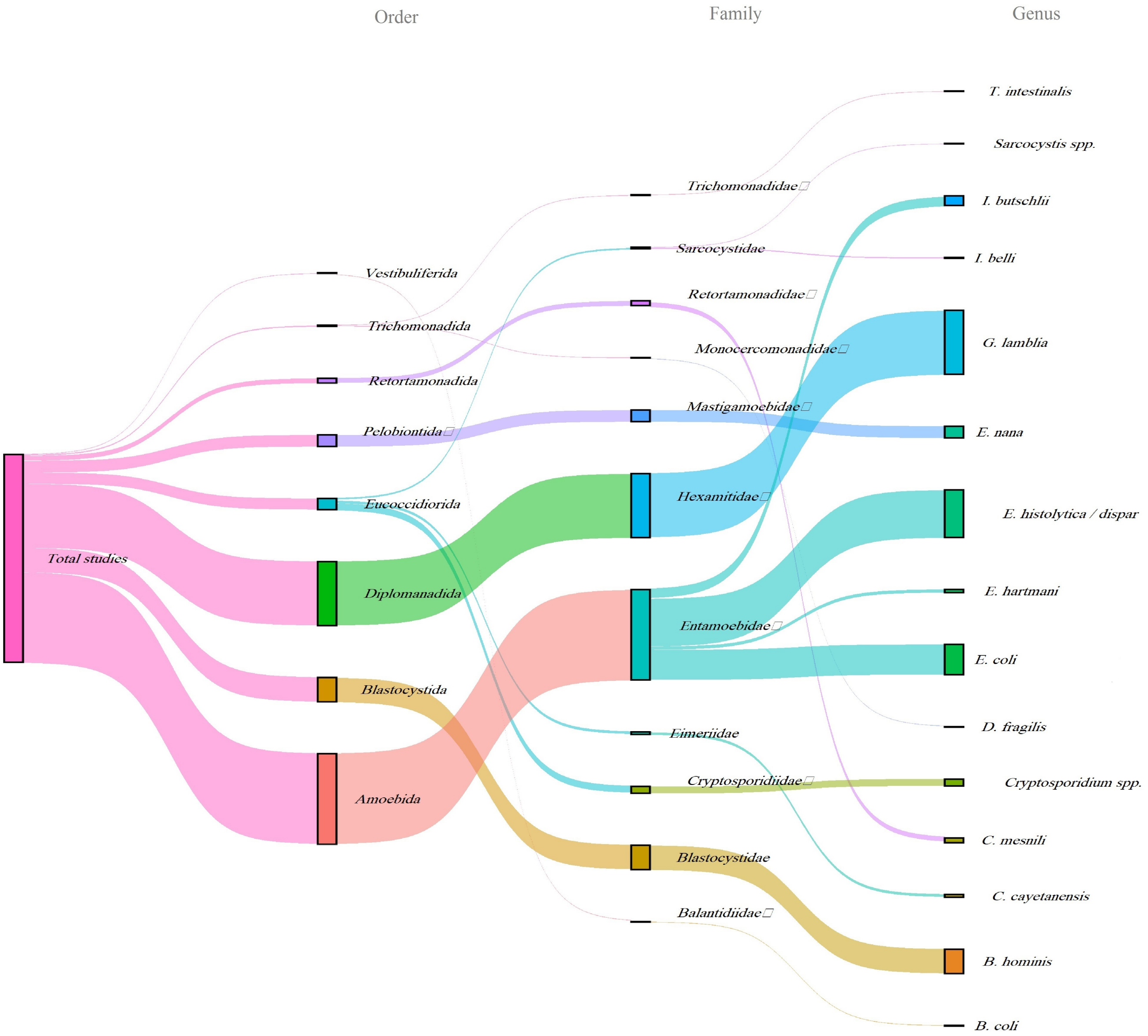

Supplementary Figure 3. Distribution of intestinal protozoan parasites among Asian schoolchildren per taxonomic order, family, and genus.
