# Supplementary material for: Prevalence of intestinal protozoan parasites among Asian schoolchildren: a systematic review and meta-analysis
Source: Infection. 2024 Jul 9;52(6):2097–133. doi: 10.1007/s15010-024-02339-1 (PMC11621188; doi:10.1007/s15010-024-02339-1)
Supplement: Supplementary file 4 — Supplementary file4 (DOCX 19 KB) [file 15010_2024_2339_MOESM4_ESM.docx]

**Supplementary Table 1.** Search terms for each database.

### PubMed: 7,764 results

| Search: ((((((((((((((((((((((((((((((((((((((((((((((((((((Asia[Title/Abstract]) ) OR (Afghanistan[Title/Abstract])) OR (Armenia[Title/Abstract])) OR (Azerbaijan[Title/Abstract])) OR (Bahrain[Title/Abstract])) OR (Bangladesh[Title/Abstract])) OR (Bhutan[Title/Abstract])) OR (Brunei[Title/Abstract])) OR (Myanmar[Title/Abstract])) OR (Cambodia[Title/Abstract])) OR (China[Title/Abstract])) OR (Georgia[Title/Abstract])) OR (Hong Kong[Title/Abstract])) OR (India[Title/Abstract])) OR (Indonesia[Title/Abstract])) OR (Iran[Title/Abstract])) OR (Iraq[Title/Abstract])) OR (Israel[Title/Abstract])) OR (Japan[Title/Abstract])) OR (Jordan[Title/Abstract])) OR (Kazakhstan[Title/Abstract])) OR (North Korea[Title/Abstract])) OR (South Korea[Title/Abstract])) OR (Kuwait[Title/Abstract])) OR (Kyrgyzstan[Title/Abstract])) OR (Laos[Title/Abstract])) OR (Lebanon[Title/Abstract])) OR (Macau[Title/Abstract])) OR (Malaysia[Title/Abstract])) OR (Maldives[Title/Abstract])) OR (Nepal[Title/Abstract])) OR (Oman[Title/Abstract])) OR (Pakistan[Title/Abstract])) OR (Philippines[Title/Abstract])) ) OR (Qatar[Title/Abstract])) OR (Saudi Arabia[Title/Abstract])) OR (Singapore[Title/Abstract])) OR (Sri Lanka[Title/Abstract])) OR (Syria[Title/Abstract])) OR (Taiwan[Title/Abstract])) OR (Tajikistan[Title/Abstract])) OR (Thailand[Title/Abstract])) OR (Timor-Leste[Title/Abstract])) OR (Turkey[Title/Abstract])) OR (Turkmenistan[Title/Abstract])) OR (United Arab Emirates[Title/Abstract])) OR (Uzbekistan[Title/Abstract])) OR (Vietnam[Title/Abstract])) OR (Yemen[Title/Abstract]) AND (English[Filter])) AND ((((prevalence[Title/Abstract]) OR (prevalence[MeSH Terms])) OR (frequency[Title/Abstract])) OR (Epidemiology[MeSH Terms]))) OR (Incidence[MeSH Terms]))) AND ((((((((((("parasitic diseases"[Title/Abstract]) OR (parasitic diseases[MeSH Terms])) OR ("parasites"[Title/Abstract])) OR (parasites[MeSH Terms])) OR ("parasitic infections"[Title/Abstract])) OR ("protozoan parasites" [Title/Abstract])) OR ("protozoan infections"[Title/Abstract])) OR (protozoan infections[MeSH Terms])) OR ("protozoan diseases"[Title/Abstract])) OR ("protozoan pathogens "[Title/Abstract]))) OR ("intestinal protozoans"[Title/Abstract])) AND ("Child, Preschool"[MeSH Terms])) OR ("Child, Preschool"[Title/Abstract])) OR ("school-age children"[Title/Abstract])) Filters applied: Full text, Humans, English, Child: birth-18 years, Preschool Child: 2-5 years, Child: 6-12 years, Adolescent: 13-18 years.  Scopus: 1,705 documents |
| --- |
| ( TITLE-ABS-KEY ( parasitic AND diseases ) OR TITLE-ABS-KEY ( parasites ) OR TITLE-ABS-KEY ( protozoan AND parasites ) OR TITLE-ABS-KEY ( protozoan AND parasite ) OR TITLE-ABS-KEY ( protozoa ) AND TITLE-ABS-KEY ( preschool AND children ) OR TITLE-ABS-KEY ( schoolchildren ) OR TITLE-ABS-KEY ( school AND children ) AND TITLE-ABS-KEY ( prevalence ) OR TITLE-ABS-KEY ( epidemiology ) OR TITLE-ABS-KEY ( frequency ) OR TITLE-ABS-KEY ( incidence ) AND TITLE-ABS-KEY ( asia ) OR TITLE-ABS-KEY ( asian ) OR TITLE-ABS-KEY ( india ) OR TITLE-ABS-KEY ( china ) OR TITLE-ABS-KEY ( indonesia ) OR TITLE-ABS-KEY ( pakistan ) OR TITLE-ABS-KEY ( bangladesh ) OR TITLE-ABS-KEY ( japan ) OR TITLE-ABS-KEY ( philippines ) OR TITLE-ABS-KEY ( vietnam ) OR TITLE-ABS-KEY ( iran ) OR TITLE-ABS-KEY ( turkey ) OR TITLE-ABS-KEY ( thailand ) OR TITLE-ABS-KEY ( myanmar ) OR TITLE-ABS-KEY ( south AND korea ) OR TITLE-ABS-KEY ( iraq ) OR TITLE-ABS-KEY ( afghanistan ) OR TITLE-ABS-KEY ( saudi AND arabia ) OR TITLE-ABS-KEY ( uzbekistan ) OR TITLE-ABS-KEY ( yemen ) OR TITLE-ABS-KEY ( malaysia ) OR TITLE-ABS-KEY ( nepal ) OR TITLE-ABS-KEY ( syria ) OR TITLE-ABS-KEY ( srilanka ) OR TITLE-ABS-KEY ( kazakhstan ) OR TITLE-ABS-KEY ( cambodia ) OR TITLE-ABS-KEY ( jordan ) OR TITLE-ABS-KEY ( azerbaijan ) OR TITLE-ABS-KEY ( tajikistan ) OR TITLE-ABS-KEY ( israel ) OR TITLE-ABS-KEY ( laos ) OR TITLE-ABS-KEY ( kyrgyzstan ) OR TITLE-ABS-KEY ( turkmenistan ) OR TITLE-ABS-KEY ( singapore ) OR TITLE-ABS-KEY ( palestine ) OR TITLE-ABS-KEY ( lebanon ) OR TITLE-ABS-KEY ( oman ) OR TITLE-ABS-KEY ( kuwait ) OR TITLE-ABS-KEY ( georgia ) OR TITLE-ABS-KEY ( mongolia ) OR TITLE-ABS-KEY ( armenia ) OR TITLE-ABS-KEY ( qatar ) OR TITLE-ABS-KEY ( bahrain ) OR TITLE-ABS-KEY ( cyprus ) OR TITLE-ABS-KEY ( bhutan ) OR TITLE-ABS-KEY ( maldives ) OR TITLE-ABS-KEY ( brunei ) OR TITLE-ABS-KEY ( united AND arab AND emirates ) OR TITLE-ABS-KEY ( timor AND leste ) ) AND PUBYEAR > 1999 AND PUBYEAR < 2024 AND ( LIMIT-TO ( LANGUAGE , "English" ) ) AND ( LIMIT-TO ( EXACTKEYWORD , "Child" ) OR LIMIT-TO ( EXACTKEYWORD , "Humans" ) OR LIMIT-TO ( EXACTKEYWORD , "Preschool Child" ) OR LIMIT-TO ( EXACTKEYWORD , "Child, Preschool" ) OR LIMIT-TO ( EXACTKEYWORD , "Adolescent" ) OR LIMIT-TO ( EXACTKEYWORD , "School Child" ) ) AND ( LIMIT-TO ( DOCTYPE , "ar" ) )  Web of science interface as follow: 1,049 recodes |
| TS=("school-age population" OR "school age" OR "preschool child" OR "school child" OR "Child" OR child* OR schoolchild* OR preschool* OR kindergarten* OR "school age*" OR schoolboy* OR schoolgirl* OR preteen* OR p?ediatr* OR student OR schoolchildren OR children OR preadolescents)  TS=(“Parasitic diseases” OR “Parasitic infection” OR parasites OR “spurious parasite” OR “parasite infection” OR “Protozoan infections” OR “Protozoan parasite” OR “Protozoan diseases” OR “Protozoan pathogens” OR “protozoal infection” OR protozoiasis OR “protozoan infection” OR “protozoon infection”)  TS=(Prevalence OR PropORtion OR Epidemiology OR Frequency OR Rate)  TS=( Asia OR Asian OR Chine OR India OR Indonesia OR Pakistan OR Bangladesh OR Japan OR Philippines OR Vietnam OR Turkey OR Iran OR Thiland OR Myanmar OR “south Korea” OR Iraq OR Afghanistan OR Saudi Arabia OR Uzbekistan OR Malysia OR Yemen OR Nepal OR “North Korea” OR “Sri Lanka” OR Kazakhstan OR Syria OR Cambodia OR Jordan OR Azebaijan OR “United Arab Emirates” OR Tajikistan OR Isreal OR Laos OR Lebanon OR Kyrgystan OR Turkmenistan OR Singapore OR Oman OR “State of Palestine” OR Kuwait OR Georgia OR Mongolia OR Armenia OR Qatar OR Bahrain OR Timor-Leste OR Cyprus OR Bhutan OR Maldives OR Brunei OR Taiwan OR ‘Hong Kong” OR Macao)  #1 AND #2 AND #3 AND #4  ProQuest interface as follow: 249 records |
| AB,TI(Asia OR Afghanistan OR Armenia OR Azerbaijan OR Bahrain OR Bangladesh OR Bhutan OR Brunei OR Myanmar OR Cambodia OR China OR Georgia OR "Hong Kong" OR India OR Indonesia OR Iran OR Iraq OR Israel OR Japan OR Jordan OR Kazakhstan OR "North Korea" OR "South Korea" OR Kuwait OR Kyrgyzstan OR Laos OR Lebanon OR Macau OR Malaysia OR Maldives OR Mongolia OR Nepal OR Oman OR Pakistan OR Philippines OR Qatar OR "Saudi Arabia" OR Singapore OR "Sri Lanka" OR Syria OR Taiwan OR Tajikistan OR Thailand OR "Timor-Leste" OR Turkey OR Turkmenistan OR "United Arab Emirates" OR Uzbekistan OR Vietnam OR Yemen) AND AB,TI(prevalence OR frequency OR Epidemiology OR Incidence) AND AB,TI(“parasitic diseases, parasites” OR “parasitic infections” OR “protozoan parasites” OR “protozoan infections” OR “protozoan diseases” OR “protozoan pathogens” OR “intestinal protozoans”) AND AB,TI(“Child, Preschool” OR ”school-age children”) |
