# Supplementary material for: Prevalence of intestinal protozoan parasites among Asian schoolchildren: a systematic review and meta-analysis
Source: Infection. 2024 Jul 9;52(6):2097–133. doi: 10.1007/s15010-024-02339-1 (PMC11621188; doi:10.1007/s15010-024-02339-1)
Supplement: Supplementary file 5 — Supplementary file5 (DOCX 38 KB) [file 15010_2024_2339_MOESM5_ESM.docx]

**Supplementary Table 2.** Quality assessment using the Newcastle–Ottawa scale modified for cross-sectional studies

| No. | First author | Year | Selection  (maximum of 5 stars) | Comparability  (maximum of 2 stars) | Outcome  (maximum of 3 stars) | Total Score |
| --- | --- | --- | --- | --- | --- | --- |
| 1 | Khudruj | 2000 | **** | ** | ** | 8 |
| 2 | LEE et al | 2000 | *** | * | ** | 6 |
| 3 | Rafiei et al | 2000 | *** | * | ** | 6 |
| 4 | Orabi | 2000 | *** | * | *** | 7 |
| 5 | Yong et al | 2000 | *** | ** | *** | 8 |
| 6 | Shahabi | 2000 | **** | ** | ** | 8 |
| 7 | Chandrasena et al | 2000 | *** | * | ** | 6 |
| 8 | Saifi et al | 2001 | ** | * | ** | 5 |
| 9 | Uga et al | 2002 | *** | * | *** | 7 |
| 10 | LEE et al | 2002 | ** | * | ** | 5 |
| 11 | Waikagul et al | 2002 | *** | ** | *** | 8 |
| 12 | Monawar Hosain et al | 2003 | *** | * | ** | 6 |
| 13 | Piangjai et al | 2003 | *** | ** | *** | 8 |
| 14 | Chhetri et al | 2003 | *** | * | *** | 7 |
| 15 | Ahmed Zakai | 2004 | **** | ** | ** | 8 |
| 16 | Nematian et al | 2004 | *** | * | ** | 6 |
| 17 | Astal | 2004 | ** | * | ** | 5 |
| 18 | Okyay et al | 2004 | *** | ** | *** | 8 |
| 19 | Krishna Sharma et al | 2004 | *** | * | *** | 7 |
| 20 | Saksirisampant et al | 2004 | ** | ** | *** | 7 |
| 21 | Daryani et al | 2005 | *** | * | ** | 6 |
| 22 | Chandrashekhar et al | 2005 | *** | * | *** | 7 |
| 23 | Sadjjadi et al | 2005 | *** | * | ** | 6 |
| 24 | Uga et al | 2005 | ** | ** | *** | 7 |
| 25 | Wongjindanon et al | 2005 | *** | ** | ** | 7 |
| 26 | Chhakda et al | 2006 | **** | * | *** | 8 |
| 27 | Kanoa et al | 2006 | *** | * | *** | 7 |
| 28 | Patel et al | 2006 | *** | ** | *** | 8 |
| 29 | Saksirisampant et al | 2006 | ** | * | ** | 5 |
| 30 | Yaicharoen et al | 2006 | *** | * | *** | 7 |
| 31 | Aksoy et al | 2007 | ** | * | ** | 5 |
| 32 | Aminzadeh et al | 2007 | ** | * | ** | 5 |
| 33 | Ngrenngarmlert et al | 2007 | *** | * | *** | 7 |
| 34 | Hafiz Mahsol et al | 2008 | ** | * | ** | 5 |
| 35 | Nematian et al | 2008 | *** | * | *** | 7 |
| 36 | Almeire et al | 2008 | ** | * | ** | 5 |
| 37 | Gyawali et al | 2009 | ** | * | ** | 5 |
| 38 | Al-Shamiri et al | 2010 | *** | * | *** | 7 |
| 39 | Aly et al | 2010 | ** | * | ** | 5 |
| 40 | Sehgal et al | 2010 | *** | ** | *** | 8 |
| 41 | Singh et al | 2010 | ** | ** | *** | 7 |
| 42 | Rayan et al | 2010 | **** | * | *** | 8 |
| 43 | Matthys et al | 2011 | *** | * | *** | 7 |
| 44 | S. Hussein | 2011 | ** | ** | ** | 6 |
| 45 | Saeed Jaeffer | 2011 | *** | ** | *** | 8 |
| 46 | Taheri et al | 2011 | *** | * | *** | 7 |
| 47 | Bhandari et al | 2011 | *** | ** | *** | 8 |
| 48 | Aher et al | 2011 | *** | ** | *** | 8 |
| 49 | Abdulsalam et al | 2012 | ** | * | ** | 5 |
| 50 | Daryani et al | 2012 | *** | ** | *** | 8 |
| 51 | Mukhiya et al | 2012 | **** | ** | *** | 9 |
| 52 | Panda et al | 2012 | *** | * | ** | 6 |
| 53 | Rostami et al | 2012 | *** | ** | *** | 8 |
| 54 | Shrestha et al | 2012 | *** | * | *** | 7 |
| 55 | Shoaib Khan et al | 2012 | *** | ** | *** | 8 |
| 56 | Al-Mekhlafi et al | 2013 | *** | ** | *** | 8 |
| 57 | Bilakshan Sah et al | 2013 | ** | * | ** | 5 |
| 58 | Singh Khadka et al | 2013 | *** | ** | *** | 8 |
| 59 | J. Lakhani et al | 2013 | **** | ** | *** | 9 |
| 60 | Kitvatanachai et al | 2013 | *** | * | ** | 6 |
| 61 | Ashok et al | 2013 | **** | ** | ** | 8 |
| 62 | Tandukar et al | 2013 | **** | * | ** | 7 |
| 63 | Sah et al | 2013 | ** | * | ** | 5 |
| 64 | Raj Tiwari et al | 2013 | *** | * | ** | 6 |
| 65 | Yadav et al | 2013 | *** | * | *** | 7 |
| 66 | Ullah et al | 2014 | *** | * | ** | 6 |
| 67 | Pradhan et al | 2014 | **** | ** | *** | 9 |
| 68 | Padmaja et al | 2014 | *** | * | ** | 6 |
| 69 | Kiran et al | 2014 | **** | ** | ** | 8 |
| 70 | Bilakshan Sah et al | 2014 | **** | * | ** | 7 |
| 71 | Jaiswal et al | 2014 | *** | ** | ** | 7 |
| 72 | Al-Delaimy et al | 2014 | *** | ** | *** | 8 |
| 73 | Pandey et al | 2015 | ** | ** | *** | 7 |
| 74 | Polseela et al | 2015 | **** | * | *** | 8 |
| 75 | Altınoz Aytar et al | 2015 | *** | * | *** | 7 |
| 76 | Bhattachan et al | 2015 | ** | ** | ** | 6 |
| 77 | Bhandari et al | 2015 | *** | ** | *** | 8 |
| 78 | Yadav et al | 2016 | ** | ** | ** | 6 |
| 79 | Shrestha et al | 2016 | **** | ** | ** | 8 |
| 80 | Sherchand et al | 2016 | *** | * | ** | 6 |
| 81 | Dhital et al | 2016 | *** | * | ** | 6 |
| 82 | Doi et al | 2016 | *** | * | *** | 7 |
| 83 | Osman et al | 2016 | *** | ** | *** | 8 |
| 84 | Arıkan et al | 2016 | *** | ** | *** | 8 |
| 85 | Nithyamathi et al | 2016 | *** | ** | ** | 7 |
| 86 | Korzeniewski et al | 2016 | *** | ** | *** | 8 |
| 87 | Khanal et al | 2016 | ** | ** | *** | 7 |
| 88 | R. Alsubaie et al | 2016 | **** | * | *** | 8 |
| 89 | Al-Mekhlaf et al | 2016 | *** | * | *** | 7 |
| 90 | Ghani et al | 2016 | ** | ** | ** | 6 |
| 91 | Zulfa et al | 2017 | *** | ** | *** | 8 |
| 92 | Turki et al | 2017 | ** | ** | ** | 6 |
| 93 | Sankur et al | 2017 | **** | ** | ** | 8 |
| 94 | Barazesh et al | 2017 | *** | * | *** | 7 |
| 95 | Sari et al | 2017 | *** | ** | *** | 8 |
| 96 | Saki et al | 2017 | ** | ** | ** | 6 |
| 97 | Rai et al | 2017 | *** | ** | ** | 7 |
| 98 | Bahmani et al | 2017 | *** | ** | *** | 8 |
| 99 | Jameel et al | 2017 | ** | ** | *** | 7 |
| 100 | Babakhani et al | 2017 | **** | * | *** | 8 |
| 101 | Jaiswal et al | 2017 | *** | * | *** | 7 |
| 102 | Tenali et al | 2018 | ** | ** | ** | 6 |
| 103 | Kyaw et al | 2018 | ** | ** | *** | 7 |
| 104 | Tandukar et al | 2018 | ** | ** | ** | 6 |
| 105 | Punsawad et al | 2018 | **** | ** | ** | 8 |
| 106 | Gopalakrishnan et al | 2018 | *** | * | *** | 7 |
| 107 | Diarthini et al | 2018 | *** | ** | *** | 8 |
| 108 | Assavapongpaiboon et al | 2018 | ** | ** | *** | 7 |
| 109 | Bansal et al | 2018 | **** | * | *** | 8 |
| 110 | Upama KC et al | 2019 | *** | * | *** | 7 |
| 111 | Rather et al | 2019 | ** | ** | ** | 6 |
| 112 | Gurung et al | 2019 | *** | * | ** | 6 |
| 113 | Bakarman et al | 2019 | **** | ** | ** | 8 |
| 114 | Lubis et al | 2019 | **** | * | ** | 7 |
| 115 | Qasem et al | 2020 | *** | ** | ** | 7 |
| 116 | Gupta et al | 2020 | *** | ** | *** | 8 |
| 117 | Alharazi et al | 2020 | ** | ** | *** | 7 |
| 118 | Afridi et al | 2021 | ** | ** | ** | 6 |
| 119 | Sari et al | 2021 | *** | * | *** | 7 |
| 120 | Sah et al | 2021 | *** | ** | *** | 8 |
| 121 | Shrestha et al | 2021 | *** | ** | *** | 8 |
| 122 | Wijayanti et al | 2021 | ** | ** | *** | 7 |
| 123 | Alharrazi | 2022 | *** | ** | *** | 8 |
| 124 | Dahal et al | 2022 | *** | ** | *** | 8 |
| 125 | Edrees et al | 2022 | *** | ** | *** | 8 |
| 126 | Edrees et al | 2022 | ** | ** | *** | 7 |
| 127 | Khan et al | 2022 | **** | * | *** | 8 |
| 128 | Salih et al | 2022 | *** | * | *** | 7 |
| 129 | AL-Mekhlafi et al | 2023 | ** | ** | ** | 6 |
| 130 | Karmacharya et al | 2023 | *** | ** | *** | 8 |
| 131 | Subhan et al | 2023 | *** | ** | *** | 8 |

*Indicates one criteria was followed, ** two criteria were followed, ***three criteria were followed, ****four criteria were followed, and ***** five criteria were followed
